# Phylogeography of the gall-inducing micromoth *Eucecidoses minutanus* Brèthes (Cecidosidae) reveals lineage diversification associated with the Neotropical Peripampasic Orogenic Arc

**DOI:** 10.1371/journal.pone.0201251

**Published:** 2018-08-08

**Authors:** Gabriela T. Silva, Germán San Blas, Willian T. Peçanha, Gilson R. P. Moreira, Gislene L. Gonçalves

**Affiliations:** 1 Programa de Pós-Graduação em Biologia Animal, Departamento de Zoologia, Instituto de Biociências, Universidade Federal do Rio Grande do Sul, Porto Alegre, RS, Brazil; 2 CONICET, Facultad de Ciencias Exactas y Naturales, Universidad Nacional de La Pampa, La Pampa, Argentina; 3 Programa de Pós-Graduação em Genética e Biologia Molecular, Instituto de Biociências, Universidade Federal do Rio Grande do Sul, Porto Alegre, RS, Brazil; 4 Departamento de Recursos Ambientales, Facultad de Ciencias Agronómicas, Universidad de Tarapacá, Arica, Chile; National Cheng Kung University, TAIWAN

## Abstract

We investigated the molecular phylogenetic divergence and historical biogeography of the gall-inducing micromoth *Eucecidoses minutanus* Brèthes (Cecidosidae) in the Neotropical region, which inhabits a wide range and has a particular life history associated with *Schinus* L. (Anacardiaceae). We characterize patterns of genetic variation based on 2.7 kb of mitochondrial DNA sequences in populations from the Parana Forest, Araucaria Forest, Pampean, Chacoan and Monte provinces. We found that the distribution pattern coincides with the Peripampasic orogenic arc, with most populations occurring in the mountainous areas located east of the Andes and on the Atlantic coast. The phylogeny revealed a marked geographically structured differentiation, which highlights a first split into two major clades: western (Monte and Chacoan) and eastern (Pampean and coastal forests). Together with AMOVA and network analysis, phylogeny revealed the existence of six well-defined lineages, which are isolated by distance. The TMRCA for *Eucecidoses* was estimated at ca. 65 Mya, and the divergence among major clades occurred by the Plio-Pleistocene ca. 20–25 Mya, with the extant six lineages emerging about 0.9 to 5.7 Mya (later than the rise of *Schinus*). These results are associated with a diversification pattern of either a late burst of speciation or early extinction. Population range expansion for some lineages concurring with major climatic changes that occurred during the wet–dry events of the Pleistocene in the region was recovered in both neutrality tests and past dynamics through time analysis. A possible biogeographic scenario reconstructed suggests that *Eucecidoses* likely emerged from a central meta-population in the south and later dispersed (ca. 38 Mya) using western and eastern as two major routes. Thus, a combination of dispersal and vicariance events that occurred in the ancestral populations might have shaped the current distribution of extant lineages. Speciation driven by host plant shift is potentially involved in the evolutionary history of *Eucecidoses*.

## Introduction

The Neotropical region is well known for its biodiversity, biome heterogeneity, geological history and the complex pattern of species distribution [1, 2]. Biogeographic hypotheses regarding the distribution of species in this highly diverse region are mostly associated with climatic and geological disturbances (*e*.*g*. tectonic events, variation in sea level and temperature) in association with periods of geographic isolation and fusion [3, 4, 5]. However, there are some conflicts regarding the historical and ecological processes responsible for the observed diversity [6]. Phylogeographic patterns responsible for shaping species diversity in South America are highly complex, forming a mosaic, and are largely unknown. Furthermore, studies have been conducted for specific areas only, as for example the Amazon, Atlantic forest, Pampas and Patagonia; little attention has been given to comparing them regarding the existence of a common faunal history (for a review, see [5]).

Although recognized as an area of 'biotic hybridization' between Neotropical and Andean regions, the South American transition zone (*sensu* Morrone, [7]) has been little explored in this regard, particularly in relation to the Argentinian mountainous systems located on the east side of the Andes (in the Prepuna and Monte provinces). These areas and the mountainous ones located in the Atlantic coast of Brazil (Parana dominion) apparently share extant lineages for a number of endemic species, a pattern supposedly determined by Tertiary tectonics [8–10]. Furthermore, it has been proposed recently that these mountainous areas were historically connected in the southernmost portion of the Pampean province from a faunistic perspective, thus forming a U-shaped distribution pattern for species involved in association with the existence of the Peripampasic orogenic arc (for a review, see [11]). However, in testing for the influence of this arc on diversity and species distributions no study has included divergence dates of the lineages involved (e.g. [11, 12, 13]). Furthermore, the influence of orogenic events on fauna diversity in those regions has not been tested for insects yet, and remains unexplored from a phylogeographic perspective at the specific level for any taxon. Here all these aspects are considered jointly to explore the existence of the orogenic arc and corresponding influence, if any, on the diversity of a gall-inducing micromoth *Eucecidoses minutanus* Brèthes (Cecidosidae), associated with *Schinus polygamus* (Cavanilles) Cabrera (Anacardiaceae).

The Cecidosidae is an ancient group of monotrysian Heteroneura micromoths [14, 15]. It is currently composed of seven genera (*Cecidoses* Curtis, *Scyrotis* Meyrick, *Dicranoses* Kieffer & Jörgensen, *Eucecidoses* Brèthes, *Oliera* Brèthes; *Xanadoses* Hoare & Dugdale, and *Cecidonius* Moreira & Gonçalves) and 19 species [14, 16, 17]. Despite hiding a putative species diversity that is yet to be discovered, cecidosids are very interesting from a geographical and ecological perspectives: 1) they have Gondwanic distribution ranges, thus limited to Southern Hemisphere (*Scyrotys* in Southern Africa, *Xanadoses* in New Zealand and the remaining genera in southern South America); 2) two highly specialized feeding habits are found (bark-mining in *Xanadoses* and gall-inducing in the other genera); and 3) particularly the gall inducers are intimately associated with their Anacardiaceae host plants (*Schinus* Linnaeus and *Searsia* F.A. Barkley, in South America and Africa, respectively). In addition, most of the South American species 4) inhabit several biogeographic provinces, 5) have localized populations determined by a patchy distribution of their hostplants, and 6) adults are short lived and supposedly do not disperse much. These characteristics make them ideal candidates for testing hypotheses related to the role of historical biogeography in lineage diversification.

**Fig 1:**
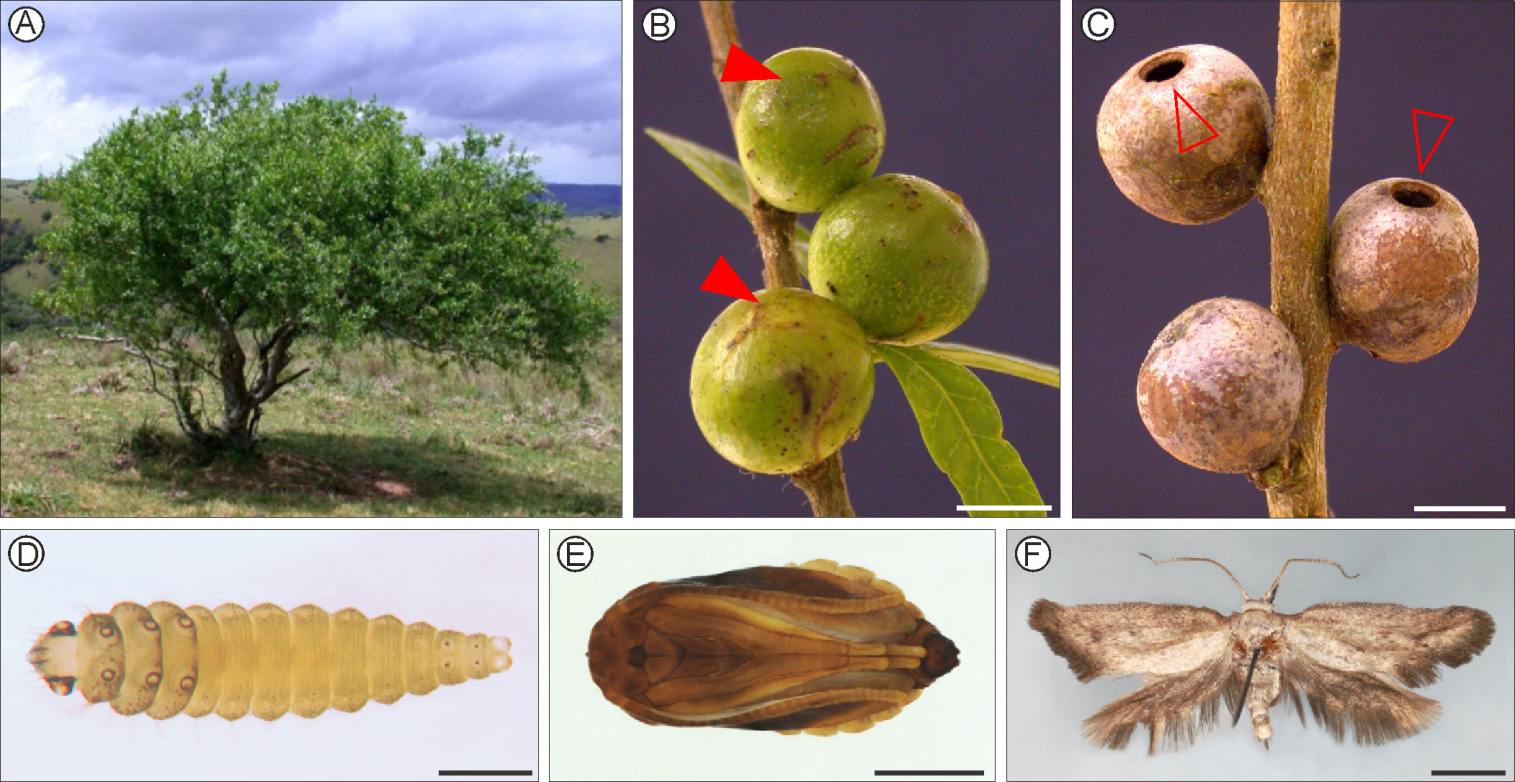
Natural history of *Eucecidoses minutanus* on *Schinus polygamus* plants from populations of the Chacoan domain, Pampean province, Brazil (Ch1_3; See Table 1). A, Isolated host plant on hilltop; B, mature galls on host plant branch (arrows point to developing opercula); C, empty, senescent galls (arrows indicate open opercula); D, last instar larva; E, pupa; F, pinned, dried adult (female, LMCI 175–34). Scale bars = 5, 5, 1, 2, 2 mm, respectively.

*Eucecidoses* in particular is ideal to be explored within such a phylogeographic scenario since it includes only one species (*E*. *minutanus*; Fig 1B–1F), uses only one highly polymorphic species as hostplant (*S*. *polygamus*; Fig 1A) and has a wide geographic distribution. It ranges from east to west through most of southern South America with scattered populations located in the South American Transition Zone, Chaco and Parana dominions that include five provinces (*sensu* Morrone [18]), thus intraspecific divergence is expected in this case. In addition to the major tectonic events mentioned above, putative vicariant processes driven by past climate oscillations during glacial and interglacial periods might have influenced the genetic diversity in *E*. *minutanus*, as predicted by the climate refuge hypothesis for Neotropical species (e.g. [19–21]). Additionally, the particular life history of these gall-inducing micromoths in association with localized host plant distributions might limit dispersal among populations and lead to differences even in the absence of physical barriers.

In this case study, we take a phylogeographic approach to understand better the evolutionary history of *Eucecidoses* in the Neotropical region. We began by mapping the historical geographic distribution of this cecidosid moth in association with that of its host plant, using material preserved in herbaria. Then we sampled specimens of extant populations of *E*. *minutanus* from most of its distribution range, which were used for DNA sequencing of mitochondrial loci. We address two main questions specifically: (1) how strong is the genetic structure in this species across distinct biogeographic provinces and (2) what is the temporal depth of the mtDNA genealogy. In addition to these questions, phylogenetic and phylogeographic data together with estimates of divergence times were used to develop hypotheses for the historical processes that have shaped lineage diversity in *E*. *minutanus*. Our results may be reconciled with two scenarios to explain current patterns found in *Eucecidoses*: (i) individuals from a centrally distributed population dispersed to colonize new areas, or (ii) a highly connected population lost connectivity with its peripheral populations and thus strengthened its genetic structure. We also estimated the areas of ancestral distribution and the events of diversification among the lineages of *E*. *minutanus* using the Bayesian Binary Method (BBM) and Statistical Dispersal–Vicariance analysis (S-DIVA). Findings are discussed in a broader context, thus being informative about Neotropical diversification in general.

## Material and methods

### Study area and sampling

**Fig 2:**
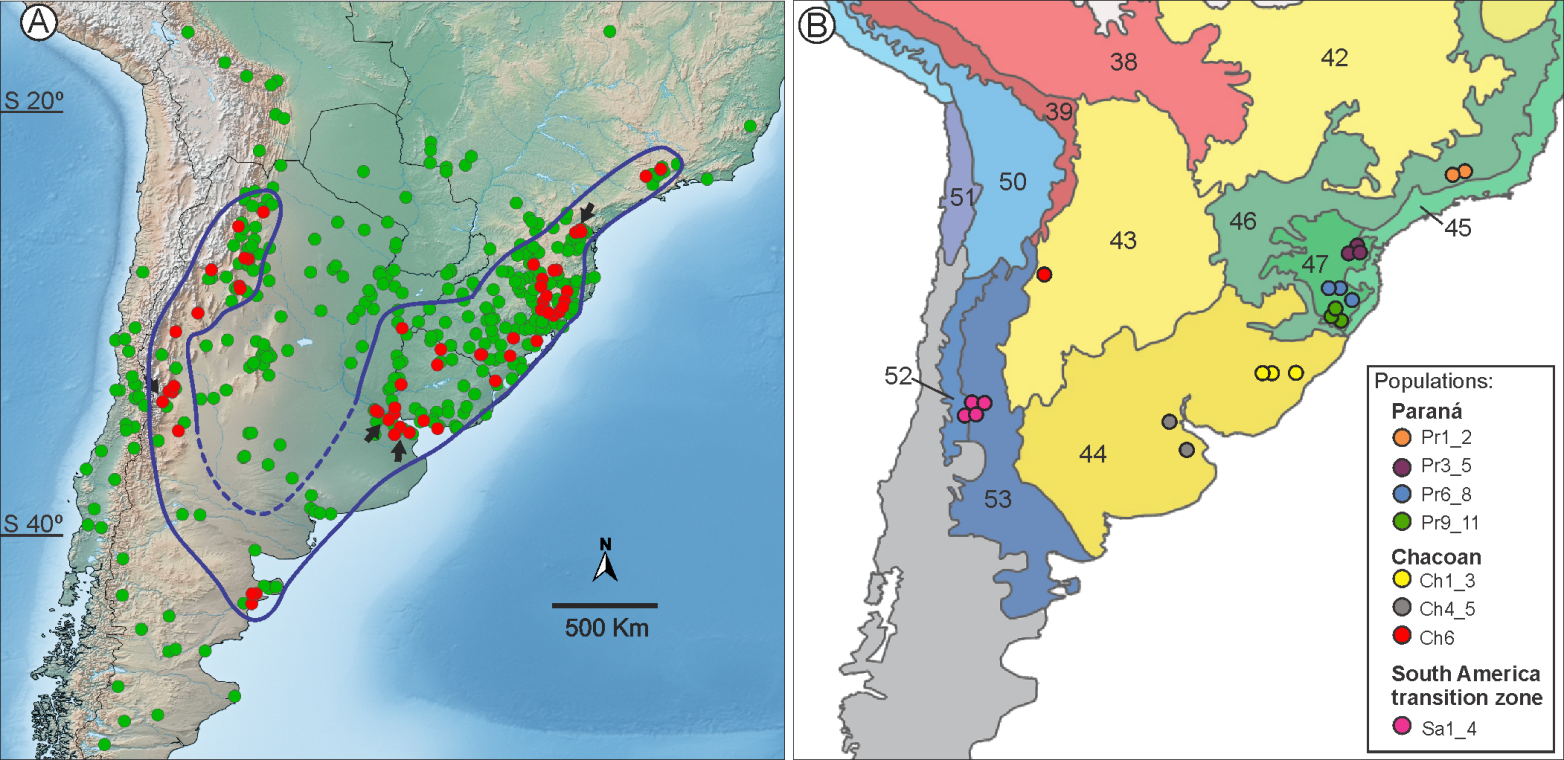
**Geographic distribution of *Schinus polygamus* (green dots) and *Eucecidoses minutanus* (red) in South America (A), and populations sampled in this study (B), according to dominions proposed by Morrone [18].** The distribution of *E*. *minutanus* is circumscribed by solid and dashed blue lines on the left map; the latter type of line represents a hypothetical section. The only four records known for *E*. *minutanus* prior to this study are indicated by black arrows. See Table 1 and S1 Appendix for complete description of localities. Areas in shades of red, green, yellow and blue on the right map represent South Brazilian (Sb), Parana (Pr), Chacoan (Ch) and South American transition zone (Sa) dominions, respectively. Numbers represent corresponding provinces: 38, Rondonia; 39, Yungas; 42, Cerrado; 43, Chacoan; 44, Pampean; 45, Atlantic; 46, Parana Forest; 47, Araucaria forest; 50, Puna; 51, Atacaman; 52, Prepuna; 53, Monte.

Prior to the beginning of this study, *E*. *minutanus* was known to exist in four populations (Fig 2A) located within the Buenos Aires (type locality) and Mendoza provinces, Argentina [22–24], and in Paraná state, Brazil [25]. These records came from either reared adults or collected galls associated with *Schinus polygamous* (Cavanilles) Cabrera (Anacardiaceae) *sensu lato*, as discussed below. These biogeographic dominions were visited from 2011 to 2014; populations of *S*. *polygamus* were progressively located and searched for the presence of *E*. *minutanus* galls. Mature galls (lignified and operculate; Fig 1B) were collected from 21 sites across three biogeographic dominions of South America (*sensu* Morrone [18]); the Parana (Pr), Chacoan (Ch) and South American Transition Zone (Sa).

**Table 1:** Characterization of *Eucecidoses* specimens used in this study. *Province*, biogeographic province; *Dominion*, biogeographic dominion (both assigned according to Morrone [18]).

Dominion	Province	Pop.	Site	Location	Lat (S); Long (W)	Vouchers	Clade
Parana							
	Parana Forest	Pr_1_2_	1	BR: São Paulo, Campos do Jordão	22° 44′ 34″; 45° 35′ 47″	LMCI 270-3A, B, C, D	Lineage 3
			2	BR: São Paulo, São Bento do Sapucaí	22° 24′ 41″; 45° 26′ 07″	LMCI 271-8A, B, C, D	Lineage 3
	Araucaria Forest	Pr_3_5_	1	BR: Paraná, Almirante Tamandaré	25° 19′ 09″; 49° 18′ 15″	LMCI 213-2A, B, C, D	Lineage 2
			2	BR: Paraná, Curitiba	25° 25′ 43″; 49° 16′ 01″	LMCI 14-51A, B, C, D	Lineage 2
			3	BR: Paraná, Campo Largo	25° 27′ 34″; 49° 31′ 38″	LMCI 214-2A, B, C, D	Lineage 2
		Pr_6_8_	1	BR: Santa Catarina, São Cristóvão do Sul	27° 16′ 01″; 50° 26′ 21″	LMCI 201-19A, B, C, D	Lineage 4
			2	BR: Santa Catarina, Curitibanos	27° 16′ 58″; 50° 34′ 51″	LMCI 205-37A, B, C, D	Lineage 4
			3	BR: Santa Catarina, São Joaquim	28° 17′ 33″; 49° 56′ 16″	LMCI 204-12A, B, C, D	Lineage 4
		Pr_9_11_	1	BR: Rio Grande do Sul, Vacaria	28° 30′ 50″; 50° 56′ 02″	LMCI 206-8A, B, C, D	Lineage 4
			2	BR: Rio Grande do Sul, Campestre da Serra	28° 47′ 34″; 51° 05′ 40″	LMCI 207-4 to 207-6	Lineage 4
			3	BR: Rio Grande do Sul, S. F. Paula	29° 26′ 45″; 50° 34′ 50″	LMCI 165-2 to 165-5	Lineage 4
Chacoan							
	Pampean	Ch_1_3_	1	BR: Rio Grande do Sul, Canguçu	31° 23′ 47″; 52° 40′ 43″	LMCI 175-3A, B, C, D	Lineage 1
			2	BR: Rio Grande do Sul, Bagé A	31° 19′ 41″; 54° 02′ 01″	LMCI 268-6	Lineage 1
			3	BR: Rio Grande do Sul, Bagé B	31° 19′ 47″; 54° 06′ 00″	LMCI 269-5	Lineage 1
		Ch_4_5_	1	AR: Buenos Aires, Zarate	34° 50′ 42″; 59° 01′ 26″	LMCI 240-36 to 240-39	Lineage 1
			2	AR: Buenos Aires, Brandsen	35° 10′ 30″; 58° 14′ 11″	LMCI 240-22,240-24,240-25	Lineage 1
	Chacoan	Ch_6_	1	AR: Tucuman	26° 43′ 57″; 65° 16′ 01″	LMCI 298-6A, B, C, D	Lineage 5
South America transition zone							
	Monte	Sa_1_4_	1	AR: Mendoza, Manzano Historico	33° 35′ 52″; 69° 22′ 56″	LMCI 240-1,240-3,240-5,240-6	Lineage 6
			2	AR: Mendoza, Cuesta Cerillos	33° 70′ 20″; 68° 55′ 01″	LMCI 197-3,197-4,197-7,197-8	Lineage 6
			3	AR: Mendoza, Lujan de Cuyo	33° 20′ 02″; 68° 52′ 56″	LMCI 163-21A, B, C, D	Lineage 6
			4	AR: Mendoza, Las Heras	32° 51′ 02″; 68° 50′ 25″	LMCI 240-14,240-17 to 240- 19	Lineage 6

Pop, general code used to identify a given population site within a biogeographic dominion.

Site, replicate (= identification number within a given population).

Location, Country, State, Municipality/political Province where samples were collected (BR = Brazil, AR = Argentina)

Coordinates, geographic coordinates (degrees, minutes, seconds—DMSo)

Vouchers, individuals collected and deposited in the LMCI = Laboratório de Morfologia e Comportamento de Insetos (UFRGS), Porto Alegre, RS, Brazil. Clade, Lineage groups defined in the phylogenetic analysis.

These sites include eight populations covering most of the range of *Eucecidoses*, corresponding to biogeographic provinces of Parana forest (Pr_1_2_), Araucaria Forest (Pr_3_11_), Pampean (Ch_1_5_), Chacoan (Ch_6_), and Monte (Sa_1_4_) (Table 1, Fig 2B). Distinct localities (up to 4, at least 50 km apart) were chosen to replicate site sampling (Table 1). Immature stages (either last instar larva or pupa) dissected from galls (eight/locality) were kept frozen at -20 ^o^C for later DNA extraction.

Field collections in Brazil were made under IBAMA/ICMBio license number 2024629, granted to G. R. P. Moreira. Specific authorization for each locality was not required since we collected galls on plants located either on road borders or private farms, with the permission of owners. None of these plants was located within or nearby protected areas, and samples did not involve endangered or protected species. Permission for field study in Argentina was approved by the Dirección de recursos naturales renovables, Mendoza (Res. 196/13 and 1109/17) and Dirección de flora y fauna silvestre y suelos, Tucumán (Res. 189/14). In Buenos Aires we collected in private lands (owners gave permission to conduct the study on sites). Field studies in Argentina did not involve any endangered or protected species. Adults were reared under room temperature in the laboratory from additional field-collected mature galls that were maintained in small plastic vials. Rearing followed the recommendations of the Animal Care and Use Committee (CEUA) of the Federal University of Rio Grande do Sul (UFRGS). After emergence they were pinned and dried, and assigned to *Eucecidoses* based on comparative analysis using wing venation and genitalia [22, 25]. Adult cecidosids are rarely represented in insect collections, which would preclude using only them in any robust biogeographic analysis. However, our preliminary observations indicated this is not the case regarding their galls existing in plant herbaria (S1 Fig). Only two cecidosid species (*Cecidoses eremita* Curtis and *E*. *minutanus*) induce external, spherical galls on *Schinus* branches [14, 22, 23]. Although similar in shape, these galls can be distinguished by their general size, wall thickness and operculum shape. Those of *C*. *eremita* are larger and have a thicker wall; they have double the diameter and thickness of those of *E*. *minutanus* ([22]; for precise dimensions, see Loetti et al., [24]). The operculum in *C*. *eremita* looks like a stopper that was described by Curtis [26] as *"…having the diameter of the inside less than that of the external surface*, *which forms a broader rim*". This characteristic allows prompt separation of these galls independently of their general size, since by contrast the operculum of *E*. *minutanus* looks like a thin, flat cover (S1 Fig).

These aspects allowed us to search for additional geographic records (and extant populations) of *E*. *minutanus* by examining the dried material of *S*. *polygamus* preserved in the main herbaria existing in the region (complete list is presented in S1 Appendix).

The presence of one or more *E*. *minutanus* galls in a given *S*. *polygamous* exsiccate was used as evidence of its presence in that locality. Conversely, the absence of such galls in exsiccates would not necessarily demonstrate its historical absence in corresponding populations, since its presence there depends on other uncontrolled factors such as the collector and curator (i.e. their own decision about whether or not to collect and include this kind of plant material (galls) in collections). We assumed that this effect would be randomly distributed in this case, and that it should be diluted by increasing the number of herbaria visited. Thus, the method we adopted here may have underestimated the frequency and boundaries of the geographic distribution of *E*. *minutanus*, but it should still be valid to infer its pattern compared to that of the host plant. In South American herbaria, *C*. *eremita* and *E*. *minutanus* galls are found on dried-preserved material belonging to *Schinus* Linnaeus, subgenus *Duvaua* (Kunth) F. A. Barkley, section *Euduvaua* F. A. Barkey, which includes ca.15 species having single leaves and spine branches [27]. The taxonomy of species of *Schinus* is confused, however; species identification is difficult—the genus is under review (C.L.S. Luz, USP, pers. com.). For some authors (*e*.*g*. [28–30]), spatial variation in corresponding populations results from phenotypic plasticity, and thus they should all be treated in this section as a single species, *S*. *polygamus*. However, for others (*e*.*g*. Barkley, [31]), only those populations located west of the Andes should be considered as belonging to *S*. *polygamus*. The remainder would be split into closely related species that are differentiated from each other by details of leaf anatomy and reproductive structures. The latter include *S*. *fasciculatus* (Griseb.) I.M. Johnst., *S*. *longifolius* (Lindl.) Speg. and *S*. *johnstonii*in Argentina [27, 31], and *S*. *engleri* Barkley in the mountainous areas of southern Brazil [32], all presumably used as host plant by *Eucecidoses*. This aspect precluded us from approaching the role of the host plant in speciation, if any, at a small geographical scale. To overcome this taxonomic limitation and make analysis from a broad geographic perspective feasible, we treated all as *S*. *polygamus* (*sensu lato*), taking into account as synonyms species listed by Cabrera [28] and Fleig [29, 30] (see S1 Appendix).

### Laboratory procedures

Total genomic DNA was extracted from tissue samples (larvae, pupae and/or adults) using the PureLink Genomic DNA Kit (Life Technologies). About 2.7 kb of mitochondrial loci were amplified via polymerase chain reaction (PCR), including the cytochrome oxidase subunit I (*CoI*), tRNALeu, cytochrome oxidase subunit II (*CoII*) and rRNA16S [33, 34]. PCR was performed using 10 ng genomic DNA, 10 pmol each primer, 1 U Taq polymerase (Life Technologies, California, USA) and 1.5 mM MgCl_2_ in a volume of 20 uL using a ABI thermal cycler (Applied Biosystems, Foster City, California, USA). The cycling parameters of the PCR, primer sequence and amplified length of each locus are described in S1 Table. PCR products were purified using the enzymatic method of Exonuclease and Alkaline Phosphatase IT (ThermoFisher Scientific, USA). The sequencing products were separated in an ABI 3730xl DNA analyzer (Applied Biosystems, California, USA). Sequences and raw sequence chromatograms were visualized, edited and aligned using CodonCodeAligner v5.1.5 (Centerville, MA, USA). All sequences generated in this study are available in the GenBank database, accession numbers: MH667739—MH667811 (*CoI*, tRNA-Leu, *CoI*) and MH667669—MH667738 (rRNA16S) (S2 Table).

### Phylogenetic analysis and divergence times

Maximum likelihood (ML) and Bayesian approaches were used for phylogenetic inference on the mtDNA-concatenated dataset. The appropriate substitution model and optimal partitioning were determined using PartitionFinder v1.1 [35]. The GTR + Gamma model proved most suitable according to the Bayesian information criterion for all markers individually as well as the combined dataset. We first reconstructed ML trees using the PhyML plugin in Geneious v.11.0.4 [36] for each genetic marker individually and assessed them for contamination issues or conflicting signals, and then we repeated that approach for the combined markers. There was no incongruence between the phylogenetic signals of different datasets. In all subsequent analyses, the dataset was analyzed following the partitioning from PartitionFinder: four partitions mostly following the division of gene fragments, as well as the second and third codon positions of *CoI*. Searches were based on 100 bootstrap replicates, followed by a thorough ML search. Clades with bootstrap values > 70% were considered as strongly supported, following Hillis and Bull [37]. Bayesian inference was performed in the BEAST v2.4.3 software [38]. Three independent Markov Chain Monte Carlo (MCMC) runs were performed, each with four streams per 50 million steps of the MCMC, sampled every 5,000 generations and discarding 5 million burn-in (about 10% of trees discarded), starting the initial trees randomly, without restriction. We assessed chain convergence by comparing the results of independent runs, and considered MCMC sampling sufficient when ESS reached > 200 for all parameters. Convergence and the effective sample size of all MCMC runs were checked in Tracer 1.6 (http://tree.bio.ed.ac.uk/software/tracer/). The software DensiTree [39] was used to draw the 8,000 tree set transparently. This allows one to evaluate properties of the tree such as well-supported clades and topological uncertainty. The tree set was processed in TreeAnnotator v1.8 (supplied with the BEAST package); a consensus tree was obtained, displayed and edited with FigTree v1.4 (http://tree.bio.ed.ac.uk/software/figtree/). The time of the most recent common ancestor (TMRCA) for relevant nodes and major mitochondrial clades was reported as the mean value of node height with 95% highest posterior density interval (HPD). Support of nodes was provided by clade posterior probabilities (BPP) directly estimated from the consensus topology. Those nodes with BPP > 0.95 were considered strong according to Erixon *et al*., [40]. The cecidosid species *Oliera argentinana* and *Cecidoses eremita* were incorporated in the analysis based on the sister relationship proposed by Moreira *et al*. [23]. The tree was rooted with representative species of Adeloidea: *Prodoxus quinquepunctellus* (Prodoxidae) and *Incurvaria masculella* (Incurvariidae) [15, 41, 42]. Divergence times were estimated in BEAST 2. For a speciation tree prior we ran the Birth-Death Process, Yule Pure Birth, Coalescent Constant Size and Coalescent Exponential Population under a comparative framework; all retrieved the same tree topology and highly similar parameters (Table 2). Plots of parameters for stationarity and for effective sample (ESS) appeared stable and with high values (>200) in all cases. We repeated the analyses to ensure that topologies were consistent between independent MCMC chains. Since the birth-death tree has successfully been used in modeling speciation and extinction—it is used as a prior distribution when inferring phylogenies using Bayesian methods [38], and this was the lowest likelihood modelled with our dataset, we chose it as the prior. We tested whether our data followed a strict or a relaxed molecular clock; then we examined the coefficient of variation of the branch rates using the lognormal relaxed molecular clock model. The coefficient of variation was 1.78 (HPD 1.47–2.02), suggesting departure from a strict molecular clock (a condition in which the coefficient of variation equals zero). Therefore, divergence times were estimated allowing branch lengths to vary under a lognormal relaxed clock [43], following a normal distribution centered on the fossil age, reflecting the bi directionality of uncertainty inherent in such calibrations [44]. We applied a calibration to set the prior on 120±10 Myr [45] for the crown clade of the Adeloidea (Cecidosidae (Prodoxidae, Incurvariidae)), using a fossil from the Early Cretaceous [46] assigned to Incurvariidae. Evolutionary relationships among families within Adeloidea are not fully resolved [42].

**Table 2:** Speciation tree priors run in Beast 2. *Eucecidoses* dataset comprised by 2,720bp (CoI = 1560 bp, CoII = 688 bp, and rRNA = 470 bp) of mitochondrial DNA sequences.

Model	LnL	TreeHeight	Kappa	Rate	
				Mean	Variance
Birth-Death	-7215.164	118.936	3.090	1.092E^-3^	1.447E^-7^
Calibrated Yule	-7228.528	119.907	3.092	1.539E^-3^	1.975E^-7^
Coalescence Constant population	-7230.097	119.961	3.091	1.128E^-3^	1.642E^-7^
Coalescence Exponential population	-7230.223	119.896	3.095	1.111E^-3^	1.898E^-7^

### Genetic and geographic structure

Standard diversity indices (number of different haplotypes, haplotype and nucleotide diversity) were estimated in the program Arlequin v3.5 [47]. This program was also used to assess the level of genetic structure (i.e., gene flow) among subpopulations using φST, which is analogous to Wright’s *F*-statistics but takes into account the genetic distance among haplotypes [47]. The influence of biogeographic scenarios (i.e. major geographic distances [western *vs*. eastern groups], dominions and provinces) in the allocation of intraspecific variation was investigated through the analysis of hierarchical levels of molecular variance (AMOVA; Excoffier *et al*. [48]) also in the program Arlequinv3.5. Pairwise genetic distance among lineages was estimated based on Kimura 2-parameter (K2P) model [49], with 1000 bootstrap of replication, and are presented in percentage.

We constructed an intraspecific genealogy using the concatenated dataset to represent evolutionary relatedness among individuals. A median-joining method [50] was implemented using the software Network 6 (http://www.fluxus-engineering.com/) to test the influence of isolation by distance (IBD) on the genetic structure of *E*. *minutanus*. The relationship between genetic and linear geographic distances of populations was evaluated using Mantel tests [51]. The significance of IBD values was assessed by the Mantel procedure (1,000 randomizations) using Arlequinv3.5.

Since historical demography might also have had an essential role in generating the variability found in *E*. *minutanus*, we performed a population expansion for each lineage. Deviations from the null hypothesis of constant population size were tested using Tajima’s D and Fu’s Fs neutrality tests [52] in the program Arlequin v3.5. Model fit to the data was tested using the sum of squared deviations (SSD) and the raggedness index (*Hri*) [53] with 1000 bootstrap replicates. We also performed a Bayesian Skyline plot (BSP) analysis, which does not assume an *a priori* growth model and infers effective population size through time based on coalescent theory [43]. The BSP was estimated in the program BEAST 1.8.4, run for 50 million iterations and sampled every 5000 steps, assuming the relaxed clock model and a normal distribution for the substitution rate, with a mean of 1.12% Myr^-1^ (Table 2) and a standard deviation of 0.13% Myr^-1^ to allow for some uncertainty in the evolutionary rate. The first 10% of the iterations were discarded to allow for burn-in. To assess the robustness of parameter estimates, two independent chains were run with identical settings. Log-files were analyzed in Tracer 1.5 (http://tree.bio.ed.ac.uk/software/tracer/) [52], and effective sample sizes were used to evaluate MCMC convergence within chains.

### Ancestral reconstruction and diversification

We reconstruct the ancestral areas and estimate diversification patterns within the genus *Eucecidoses*. Historical biogeographic processes likely shaped the current distribution of lineages and we used this approach to infer current patterns and to test whether extant populations derived from an ancestral species that colonizes novel areas (dispersal), or diverged from ancient geographical barriers (vicariance). We thus used two approaches for ancestral character reconstructions: Statistical Dispersal-Vicariance Analysis (S-DIVA) v 2.0 [54, 55] and the Bayesian Binary MCMC method (BBM) [56] as implemented in RASP v.3.0 [57]. The RASP (Reconstruct Ancestral State in Phylogenies) is a tool to reconstruct evolutionary histories using Bayesian phylogenetic inference, which reveals the probability of each possible range of an ancestral area to each node based on previously indicated biogeographic areas. This method consists of using all generated trees from the MCMC output and calculates the average frequency of an ancestral range at a node in ancestral reconstructions. The BBM method was performed using 500,000 generations with 10 chains, sampling every 100 generations; the first 500 trees were eliminated (burn-in). Fixed JC (Jukes-Cantor) were used. We defined five areas to assess the historical biogeography of the *Eucecidoses*: (A) Parana Forest, (B) Araucaria Forest, (C) Pampean, (D) Chacoan and (E) Monte, based on the classification of provinces proposed by Morrone [18].

The chronogram obtained by BEAST analysis was used to construct semi-logarithmic lineages through time (LTT) plots in TRACER version 1.5. To examine confidence in the estimated dating, confidence intervals (95%) were estimated using 2000 trees from the pool of converged Bayesian trees in the BEAST analysis. To test for significant departures from the constant speciation rate model we used the γ-statistic [58]. The gamma statistic of Pybus and Harvey [58] relates to the distributions of internode distances through time, and under the pure birth model follows a standard normal distribution with a mean of zero.

## Results

### Phylogenetic analysis

**Fig 3:**
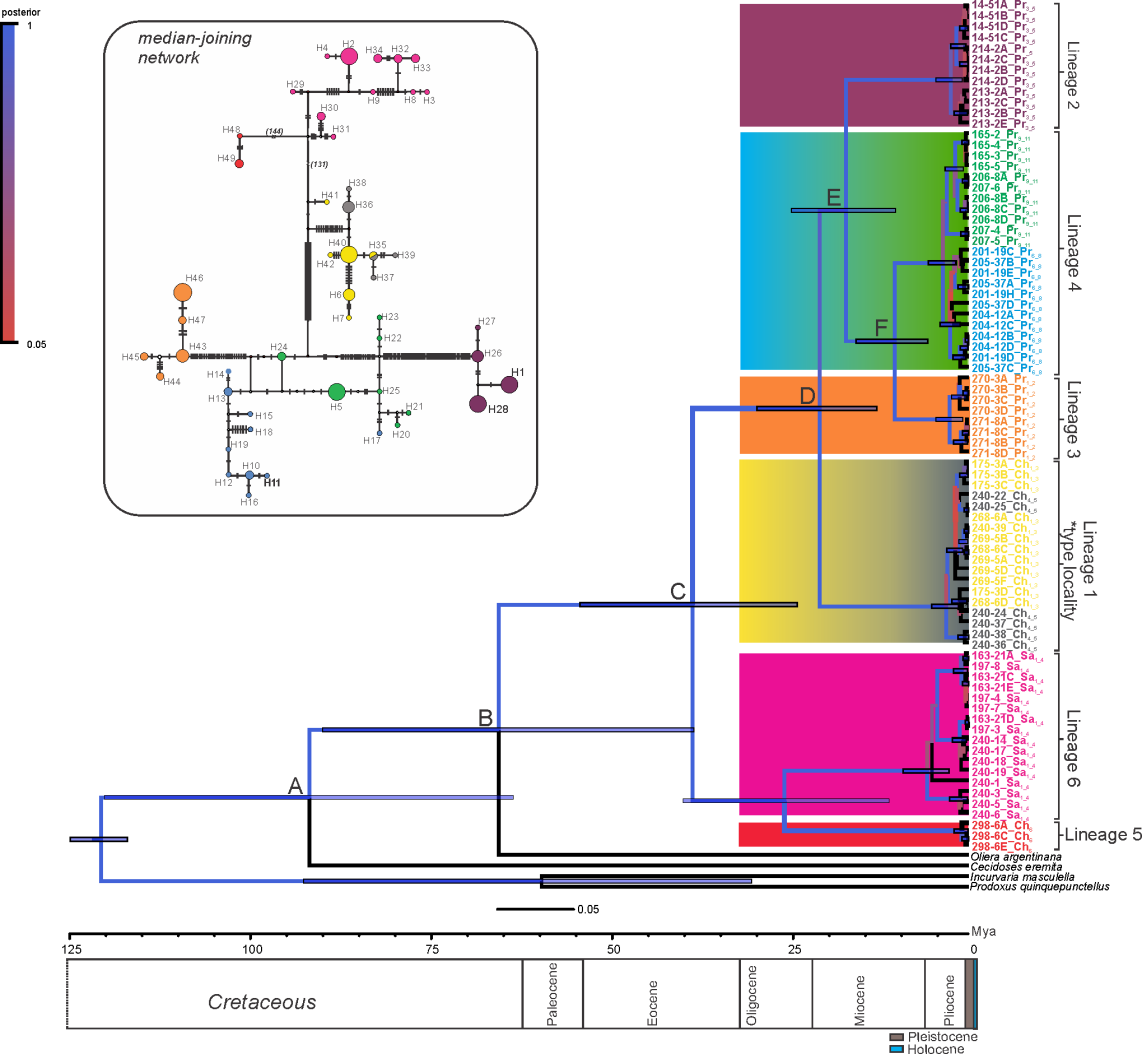
Evolutionary relationships within *Eucecidoses minutanus*. Phylogenetic tree under the relaxed uncorrelated lognormal clock Bayesian analysis reconstructed based on 2.7 Kb of mitochondrial sequences using birth-death tree prior. Capital letters at nodes (A-F) indicate major clades referred to in Table 4. Populations are indicated by the colored terminals (See Table 1 for further description). Colored squares represent the six lineages inferred; those that encompass more than one population are indicated by a gradient of colors. Posterior probabilities are indicated by the colored branches and the legend inside the figure. Mean time to the most recent common ancestor is indicated in the middle of the branches, and 95% credibility intervals (95% HPD) are represented by solid blue bars, in millions of years (Mya). The ruler at the bottom represents the rate of substitution per site. A median-joining network of haplotypes found in all populations in presented on left top. Corresponding bars along branches represent mutational steps. Circle size is proportional to haplotype frequency. Small red circles represent median vectors.

**Fig 4:**
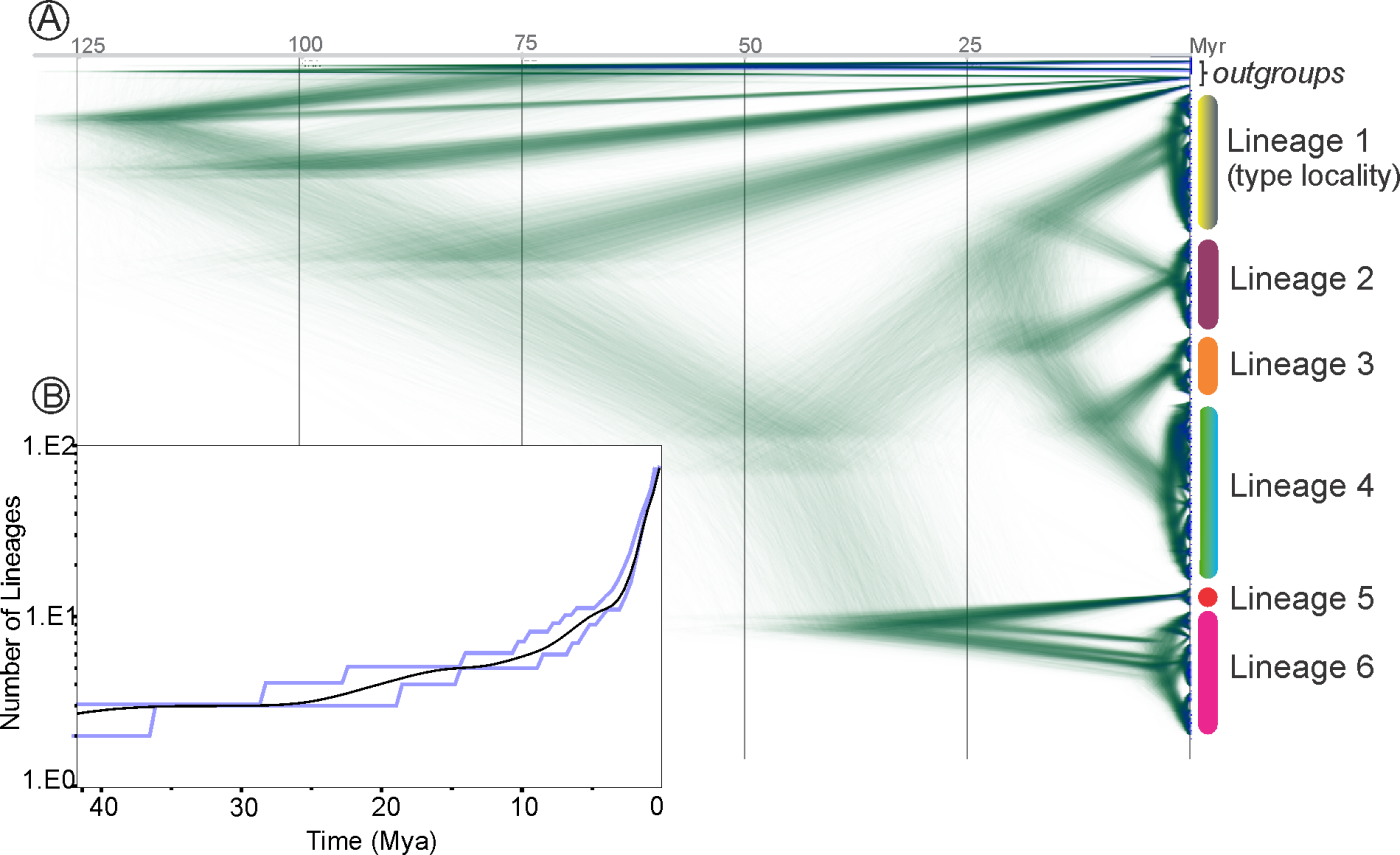
Evolutionary diversification in *Eucecidoses minutanus* resulting from Bayesian analysis based on 2.7 Kb of mitochondrial sequences. A, Densitree, a consensus posterior density of trees representing the entire posterior distribution of populations (after 8,000 trees of burnin) from the BEAST analysis. Areas where species trees agree on topology and/or branch lengths are densely colored. Variation in timing of divergences (in Myr) is shown as fuzziness along the *x*-axis. All nodes have support values > 0.99. The six clades found in the phylogenetic reconstruction (see Fig 3) are indicated by the color bar on the right, defined as lineage 1 to 6 (see Fig 3 for details). B, Lineage through time (LTT) plot (black line) highlighting a late speciation (or early extinction) pattern of diversification. Confidence intervals (95%), estimated using 2000 trees from the pool of converged Bayesian trees in BEAST analysis, are also indicated (blue lines).

The total dataset comprised 2,720bp (CoI = 1560 bp, CoII = 688 bp, and rRNA = 470 bp). The alignment was straightforward for protein-coding genes, as no internal stop codons were detected. Bayesian and ML analyses revealed the same topology with six strongly supported clades (hereafter defined as lineages 1 to 6) with marked phylogeographic structure. Since we also estimated divergence time, we selected the Bayesian tree as the basis for discussions (Figs 3 and 4). Lineage 1 is composed of populations Ch_1_3_ + Ch_1_5_ from the Pampean province, which covers the type locality and therefore the original description of *E*. *minutanus*. Novel monophyletic groups found within *Eucecidoses* are represented by lineages 2 to 6 (Figs 3 and 4). The South American Transition Zone presented only one (lineage 6), while the Chacoan and Paraná dominions showed two (lineages 1 and 5) and three lineages (lineages 2, 3 and 4), respectively. We found highly divergent lineages in the Pampean (1 [Ch_1_5_]) and Chacoan provinces (5 [Ch_6_]), both within the Chacoan dominion (Table 3). Similarly, within the Paraná dominion (Araucaria and Paraná Forest) we found lineages 2 [Pr_3_5_], 3 [Pr_6_8_ + Pr_9_11_] and 4 [Pr_1_2_], with different levels of divergence. The K2P genetic distance between these six lineages ranged from 2% to 11% (Table 2). Within each lineage variability varied from 0.1% to 1%.

**Table 3:** Genetic divergence among six lineages of *Eucecidoses* based on 2.7 Kb of mitochondrial sequences. Above diagonal: pairwise phi (ST) values based on haplotype frequency only, which indicates low rates of gene flow among all lineages. All comparisons were statistically significant; *P* ≤0.05). Below diagonal, pairwise K2P distance (numbers in brackets indicate diversity within populations). See Fig 3 for detailed description of lineages.

Lineage	1.	2.	3.	4.	5.	6.
1.	[1.2%]	0.8539	0.7989	0.7766	0.9153	0.8955
2.	4.3%	[0.2%]	0.9577	0.8839	0.9850	0.9358
3.	3.4%	4.0%	[0.2%]	0.7581	0.9816	0.9259
4.	3.5%	4.0%	1.9%	[0.6%]	0.9417	0.9157
5.	8.5%	8.7%	8.2%	8.4%	[0.08%]	0.9230
6.	10.7%	10.8%	10.0%	10.1%	9.4%	[0.1%]

**Table 4:** Estimates of the time to the most recent common ancestor (TMRCA) for the main nodes recovered in the phylogenetic reconstruction of *Eucecidoses* using 2.7 kb of concatenated mitochondrial sequences. Clade letters are indicated in the Fig 3. populations included in each lineage are presented in brackets (for details see Table 1).

Clade	TMRCA (Mya)	95% HPD
A	91.09	62.92-119-47
B	64.98	37.94–89.32
C	38.15	23.7–53.73
D	20.56	12.69–29.32
E	16.97	10.1–24.47
F	10.24	5.67–15.54
**Lineage** 1 [Ch_1_3 +_ Ch_4_5_]	3.1	1.37–5.11
**Lineage** 2 [Pr_3_5_]	2.48	0.87–4.5
**Lineage** 3 [Pr_1_2_]	2.63	0.8–4.54
**Lineage** 4 [Pr_6_8_+ Pr_9_11_]	3.64	1.74–5.61
**Lineage** 5 [Ch_6_]	0.91	0.1–1.98
**Lineage** 6 [Sa_1_ + Sa_2_4_]	5.72	2.69–9.08

### Estimation of divergence times, biogeography and diversification analyses

The mtDNA substitution rate observed in *Eucecidoses* was slow, ca. 1% per Myr (Table 2). Divergence time estimate indicated that the TMRCA of *Eucecidoses* split back in the Paleocene, ca. 65 Mya (95% HPD: 37.94–89.32) (Fig 3). The initial diversification event occurred ca. 38 Mya (95% HPD: 23.70–53.73) giving rise to two major clades. The first one diversified ca. 25 Mya (95% HPD: 10.95–39.47) and is formed by lineages 5 and 6 in the Chacoan (Ch_6_) and Monte (Sa_1_4_) provinces, respectively; the second major clade split around 20.5 Mya (95% HPD: 12.69–29.26), and originated lineages 1 to 4 in the Parana and Araucaria forests and Pampean provinces (Fig 3). The clade that includes groups from the forest provinces (lineages 2, 3 and 4) emerged around 17 Mya (95% HPD: 10.10–24.47). Although most of these clades emerged in the Paleogene Period, the date estimated for each of six particular lineages is quite recent as the Late Pliocene, including the Quaternary period (<3 Mya) (Table 4; Fig 4).

**Fig 5:**
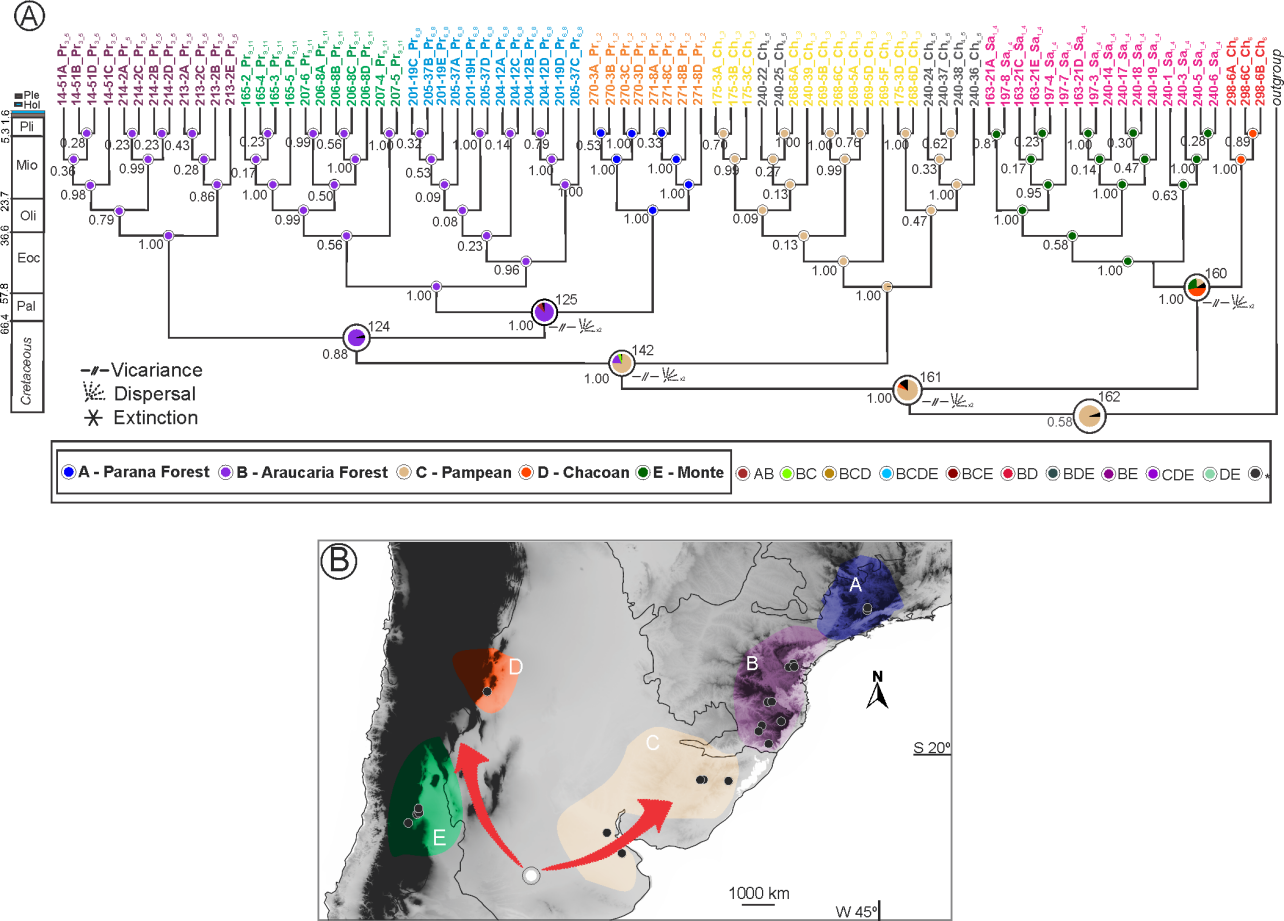
Ancestral area reconstruction using BBM analysis for *Eucecidoses* lineages considering five biogeographic groups. (A) Graphic output of ancestral distribution at each node of the Bayesian phylogeny of *Eucecidoses minutanus*. The tips indicate populations sampled (see Table 1 for details), while colored nodes represent geographic area estimated (indicated by the legend at the bottom of the figure). Circle sizes at nodes refer to frequency of ancestral range occurrence, with values indicated below the branch. Events of dispersal and vicariance, and extinction are indicated by graphic symbols B, Depiction of the historical biogeography scenario estimated; large circle and associated arrows refer to likely center of origin and colonization pathways.

The oldest lineage is 6 from the Monte region near the Andes Mountains, and the youngest ones are found in the forest provinces (lineages 2, 3 and 4), except lineage 5 from the Chacoan. A marked geographic structure was evident within the Paraná domain. Lineage 2 was divergent from the others within the same province in ca. 4%). Results from the BBM analysis suggest a complex biogeographical history in which both dispersal and vicariance have been important in shaping the current distribution pattern in *Eucecidoses* (Fig 5).

Lineages 5 and 6 indicated by the phylogeny are derived from the DE area (Chacoan + Monte). Thus a vicariance event is evident at this node, resulting in the present Chacoan (D) and Monte (E) populations. For lineages 1 to 4 the BC area is suggested (Araucaria Forest + Pampean) as a likely origin of these groups. From this node, events of vicariance and dispersal are suggested. Accordingly, lineage 1 (Pampean province [C]) might have diverged from the others through a vicariance event. Other lineages (2 to 4) shared area B (Araucaria forest) as ancestral, thus from a dispersal event lineages probably dispersed and occupied new areas through provinces (B) [Araucaria forest] and AB [Parana and Araucaria forest]. Later, there was likely a vicariance of lineages 3 and 4, between A (Parana forest) and B (Araucaria forest) areas.

Node 161 in Fig 5 represents all *Eucecidoses* lineages, suggesting that the ancestor originated in area C (Pampean), with a marginal probability of 100%. Similarly, as in the S-DIVA analysis, dispersal events associated with vicariance are evident in the common ancestor node of *Eucecidoses*. However, node 160 is ambiguous, and ancestral reconstruction for lineages 5 and 6 could not be determined. There are three possibilities: D (marginal probability of 42.22%), E (25.56%) and C (21.29%). Dispersal events associated with vicariance are also suggested by this node. The ancestral reconstruction at node 142 that represents members of lineages 1 to 4 postulates that the ancestors probably originated in the C (Pampean) area, with 79.38% marginal probability. Dispersion events associated with vicariance are evident in this node. In addition, as indicated in the S-DIVA analysis, linage 1 split from the other three lineages. Node 124 concentrates the other lineages present in the provinces of Parana forest (A) and Araucaria forest (B). Lineage 2, as well as the ancestral node 124 (with high marginal probability, 94.65%) remained in the province Araucaria forest zone. Finally, node 123 presents dispersion associated with vicariance. However, despite a marginal probability of 87.97% for Araucaria forest as the ancestral area of lineages 3 and 4, the dispersal from B (Araucaria forest) to (A) Parana forest is evident, suggesting that once the species reached a new area (through dispersion), the lineages split. The different sets of the analyses did not change the historical distribution patterns of ancestors, except that BBM analysis and S-DIVA suggest different ancestral ranges at basal nodes (S2 Fig). The maximal S-DIVA value determining support for ancestral range was 1752.48.

The lineage accumulation plot shows smooth increases in the number of lineages from the Late Pliocene (Fig 4B). The γ-statistic showed a positive value (γ = 1.941), rejecting the model of constant diversification rate (*p* = 0.02), and suggests accelerated diversification rate in the recent history of *Eucecidoses*.

### Genetic differentiation and demographic changes

High levels of genetic variability were observed in the six lineages found (Table 5).

**Table 5:** Genetic variability of six *Eucecidoses* lineages based on 2.7 Kb of mitochondrial sequences. See Table 1 for detailed description of populations.

						Neutrality test			
Lineage [Population(s)]	N	S	H	Hd	Pi	Tajima’s *D*	Fu’s *Fs*	SSD	Raggedness index
1 [Ch_1_3 +_ Ch_4_5_]	13	91	12	0.987±0.03	0.0113±0.0020	-0.018	0.088	0.0447	0.0309
2 [Pr_3_5_]	11	20	10	0.982±0.04	0.0025±0.0003	-0.115	-3.473*	0.0214	0.0492
3 [Pr_1_2_]	8	12	7	0.964±0.07	0.0071±0.0016	0.598	-1.826	0.0510	0.0982
4 [Pr_6_8_+ Pr_9_11_]	22	66	16	0.970±0.02	0.0082±0.0012	-1.017	-1.501*	0.0211	0.0536 *
5 [Ch_6_]	3	5	3	1.00±0.272	0.0035±0.0035	-	-0.077	0.2312	0.6666
6 [Sa_1_4_]	17	251	16	0.993±0.02	0.0005±0.0073	-1.935*	-1.120*	0.0220	0.0325

N, number of specimens

S, variable sites

H, number of haplotypes

Hd, haplotype diversity (±standard error)

Pi, nucleotide diversity (±standard error)

Neutrality tests using Tajima’s D and Fu’s Fs. SSD and the raggedness index were used for statistical support

*P indicates significance < 0.05

**Fig 6:**
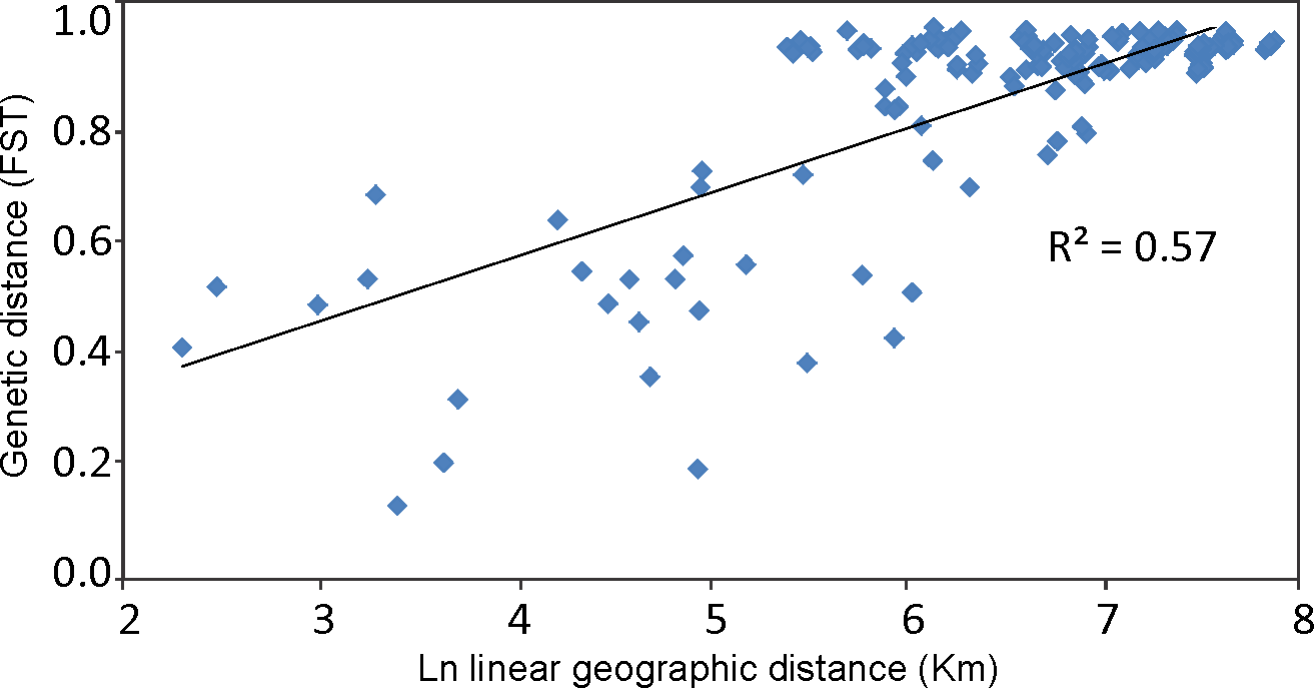
Isolation by distance plots of pairwise values for log geographic distance and genetic distance across collected sites of *Eucecidoses*. Genetic distance is given by φ-statistics (φST). Geographic distance (linear) is given in km. Statistical significance was assessed using the Mantel test (r = 0.57, p<0.001).

The number of haplotypes varied from 2 to 17 per lineage, with a total of 47 (Fig 3). Nucleotide diversity was highest in lineages 1 (Pampean) and 5 (Monte). Haplotypes are clearly geographically structured, except for lineages 4 and 1 that shared Pr_6_8_ /Pr_9_11_ and Ch_1_3_ /Ch_4_5_ haplotypes among distant populations (Fig 3). AMOVA results showed that almost half of the variation is allocated among groups (48.07%) when eastern and western groups are defined, indicating that highly divergent populations were clustered together (Table 6). Differentiation was comparatively slightly lower among groups (29.46%) when provinces and dominions were defined *a priori* but higher among populations within groups (61.4%), suggesting differentiation due to the heterogeneity of physiognomies (Table 6). The lowest portion of variance was found within populations of each biogeographic subdivision, and a non-significant difference was observed in both biogeographic groups. The genetic differentiation based on the fixation index revealed high estimates (*F*st = 0.61, *p*<0.05) for most comparisons, indicating some level of gene flow among the six lineages (Table 3).The IBD analysis indicated significant correlation between genetic and linear geographic distance (r = 0.50, p<0.001) of the collection sites (Fig 6).

**Table 6:** Analysis of Molecular Variance (AMOVA) using ɸ-statistics based on concatenated mitochondrial sequences (2.7 Kb) for lineages of *Eucecidoses* (defined on Bayesian phylogeny, see Fig 3) considering distinct geographical scenarios.

				Variance*		
#	Level	# Groups	Definition	Va	Vb	Vc
i)	Major geographic distance	2	Western [L5+L6] *vs*. Eastern [L1+L2+L3+L4]	48.07% ɸ_*ST*_ = 0.48 (*p* = 0.072)	44.51% ɸ_*CS*_ = 0.85 (*p* = 0.000)	7.43% ɸ_*CT*_ = 0.92 (*p* = 0.000)
ii)	Biogeographic dominions	3	Chacoan [L1+L5], Parana [L2+L3+L4], South America Transition Zone [L6]	29.46% ɸ_*ST*_ = 0.29 (*p* = 0.116)	61.42% ɸ_*CS*_ = 0.87 (*p* = 0.000)	9.12% ɸ_*CT*_ = 0.90 (*p* = 0.000)
iii)	Biogeographic provinces	5	Pampean (L1); Araucaria Forest (L2+L4); Parana Forest (L3); Chacoan (L5); Monte (L6 [Sa_1_4_])	34.03% ɸ_*ST*_ = 0.34 (*p* = 0.235)	56.32% ɸ_*CS*_ = 0.85 (*p* = 0.000)	9.64% ɸ_*CT*_ = 0.90 (*p* = 0.000)

* Source of variation

ɸ_ST_, Among groups (major lineages or regions)

ɸ_SC_, Among populations within groups

ɸ_CT_, Within populations (both regional and individual-population levels).

**Fig 7:**
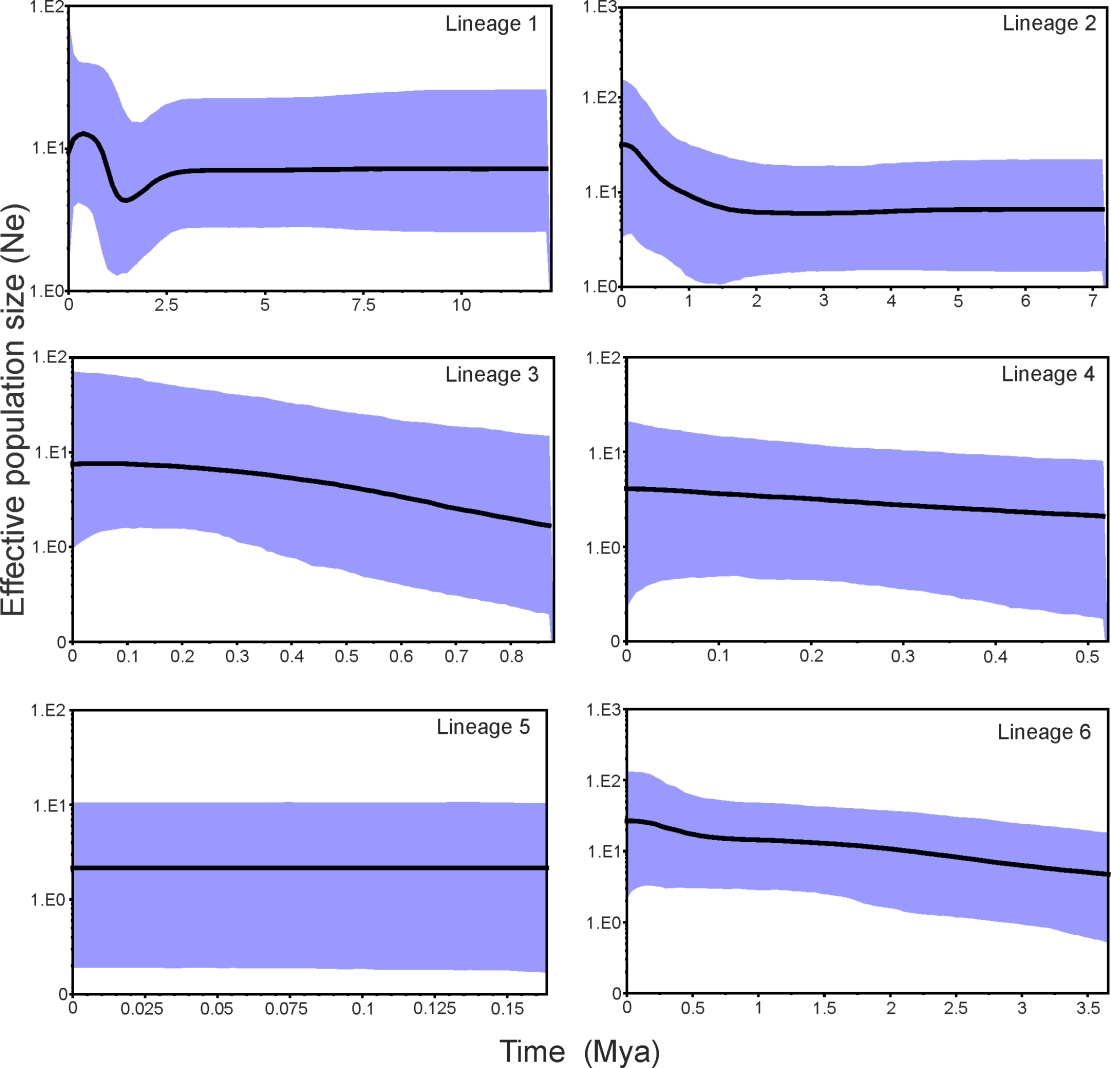
Bayesian Skyline Plots illustrating the demographic history pattern of six lineages of *Eucecidoses* along evolutionary time. The x-axes represent time in millions of years and the y-axes correspond to the product of effective population size and generation time in millions of years. The upper and lower purple shadows represent the 95% HPD and black line indicates mean population size.

Tajima’s D-test and Fu’s Fs-test yielded non-significant results in most of the lineages. Neutrality tests results were significantly negative in four populations (Table 5), which suggests an excess of low frequency variants. Since positive selection can be neglected, we infer that the significantly negative values of the neutrality test could arise from demographic expansion. This is in agreement with recent range expansion evidence during the Pleistocene as shown by BSP plots (Fig 7).

Coalescent-based inference of demographic history through BSP indicated congruent responses among lineages. A history of stability of ancestral population sizes across the evolutionary past was observed, with relatively narrow confidence intervals (Fig 7). Recent growth around 0.5 Myr for lineage 1 and 6 is supported. BSP indicated variation in effective population size among lineages, and differences in their timing of expansion. This implies some level of differences in population trajectories, particularly with respect to topography and microclimate. However, the broader confidence intervals around most recent evolutionary nodes suggest that these inferences should be made with caution.

## Discussion

### Geographic distribution

The 54 additional geographical records provided in this study demonstrated that the distribution of *E*. *minutanus* is concentrated in the mountainous systems of central and northern Argentina, east of the Andes (Monte province of the South American transition zone and Chacoan dominion/province), and southern Brazil (Araucaria and Parana forest provinces of the Parana dominion). Thus, based on records presented herein its distribution range was extended north in these mountainous regions to the Tucumán and Campos do Jordão areas, respectively in Argentina (west) and Brazil (east). Additional populations were also found in the southernmost Pampean province, within the Chacoan dominion, in Rio Grande do Sul State (Brazil), Uruguay and the Buenos Aires area (Argentina), thus forming a U-shaped distribution pattern, inferred provisionally in connection with those located in the Mendoza area. Efforts to localize extant populations of *E*. *minutanus* south of these regions in Argentina were unsuccessful. We predict that herbarium records from Chubut (Argentina) represent relict populations of *E*. *minutanus*, which should be further explored. Despite the existence of several populations of *S*. *polygamus* in the central areas located further north, we found no records for *E*. *minutanus* within the remaining Pampean and Chacoan provinces. Interestingly, species from other cecidosid genera such as *Cecidoses* Curtis and *Dicranoses* Kieffer & Jörgensen are known to occur within this area, for example in the Espinhal [59]. *Cecidoses* is the closest related genus to *Eucecidoses* [17], and uses the same host plant in this area. The absence of *E*. *minutanus* was also noted from the Patagonia area, where at least one species of *Cecidoses* is found [60]. Furthermore, *Eucecidoses* seems to have never occurred west of the Andes. *Schinus polygamus* is relatively abundant in the central regions of Chile, where in recent years we have intensively searched for cecidosids. Gall-inducing micromoths are represented there by undescribed species belonging to different genera, all associated with *S*. *polygamus*. However, they are not closely related to *Eucecidoses*, and will be treated elsewhere (for further discussion, see Moreira *et al*., [17]). Thus, we provided strong evidence that extant distribution pattern of *E*. *minutanus* cannot be explained by variation in distribution of the host plant, but it is instead related to historical geographic events.

In association with the phylogenetic results, our study gives also strong support for the existence of a common diversification history for the two main extant *E*. *minutanus* lineages that are isolated in distribution in the western and eastern mountain systems. This pattern has already been found for other arthropods, including scorpions [21], freshwater crabs [13] and spiders [11]. However, as already mentioned, these studies were not conducted within species, but in higher taxonomic categories (genus and/or family), and were not based on estimates of divergence times between lineages. Here, using a phylogeographic approach jointly with a Bayesian divergence time estimation we estimated that two *Eucecidoses* moth lineages started to diverge in these mountainous systems, ca. 38 Mya. Thus, divergence might have occurred concurrently in time with fragmentation of the corresponding peripampasic orogenic arc, due supposedly to Tertiary tectonics associated with the uplift of the Andes. This event not only might have led to the divergence of these two clades, separating them geographically, but also maintained the western one (central and northern Argentina) restricted to east of the Andes.

### Cryptic diversity and taxonomic implication

The molecular phylogeny of *Eucecidoses* presented here revealed a strong genetic structure, which showed a deep evolutionary history for mtDNA haplotypes. We found six well-supported lineages, that is, five new phylogenetic units in addition to the single one *E*. *minutanus* currently recognized. The criteria used here to distinguish lineages (i.e., phylogenetic units), in addition to reciprocal monophyly, was the threshold of genetic distance >2% that has been used to separate species in other micromoths, for example 1.39–2.37% in gracillariids [61]. We also considered as a cut off the inter-lineage distance (2 to 10%) being two times higher than intraspecific divergence (0.08%-1.2%) observed in our data. Whether all the six phylogenetic units found in this study should be described as distinct species remains as an open question that requires availability of morphological data to be addressed. Preliminary observation showed that adults of the two major clades (east *vs*. west groups) present stable differences in wing venation, pupal and larval integumentary morphologies. Additionally, within these clades males of each lineage may differ in their genitalia. This is the case for specimens of lineage 2 (Curitiba, Brazil) compared to those reared from the others (lineage 3 and 4) located in the east (Parana dominion), which might represent geographic structure of the same taxon. A separate publication with at least two of such lineages described as a different species, in conjunction with a taxonomic revision of *E*. *minutanus* will be further proposed.

The genetic differentiation found might be attributed either to isolation by distance or to marked heterogeneity of physiognomies and ecological characteristics of each region. These pathways are not necessarily mutually exclusive, and should be explored further. Despite variation in flight ability, for most lepidopteran species this is the main form of dispersal [62]. However, *Eucecidoses* is expected to present a very low dispersion capacity; like other cecidosids they may restrict their movement solely around their host plants, searching for mates and oviposition sites [63, 15]. Adults of all cecidosids have atrophied mouth appendages, and thus supposedly do not feed and live for a short time. Those of *Dicranoses capsulifex* Kieffer and Jörgensen reproduce and die in the same day of emergence [64]. These low dispersion capabilities in addition to the genetic distance observed among across provinces reinforce that populations of *Eucecidoses* can be considered different lineages, as restricted gene flow was observed, and thus fixed differences at the genotypic level.

### Time of diversification and historical biogeography

Slow nucleotide substitution rates have been found in other basal Lepidopterans [45]. The divergence rate found in *Eucecidoses* was ca. 1% per My, lower than the ‘standard’ 2.3% estimated for mtDNA in insects [65]. This likely results from old calibration point used here and by not assuming a linear relationship of sequence divergence over this period, considering the higher rate of diversification in recently split lineages. The rate of substitution (non-synonymous) is generally considered a decreasing function of effective population size [66]. Accordingly, values of genetic diversity and demographic changes in *Eucecidoses* suggest important differences in population dynamics between biogeographic regions. Populations from the central part of the distribution are more variable and have had larger population size changes, although the credible intervals are broad since only mtDNA sequences were used in this analysis [67]. The population expansion that affected groups from the central part of the distribution is roughly coincident with the origin of several subclades within the forest lineage. Given that there is no haplotype sharing and that all subclades are restricted to a single geographic population, it may be inferred that several suitable areas were colonized (or re-colonized) during this expansion, with genetic drift and population isolation later restricting each population.

Divergences leading to extant Cecidosidae genera occurred primarily in the Cretaceous; however, diversification within each genus has mainly occurred in the Cenozoic [17]. Accordingly, molecular analyses suggest that *Eucecidoses* originated during the early Paleogene, more than 60 Mya, and since its origin has experienced a long period (± 25 Mya) without speciation. This pattern might result from widespread extinctions caused by the Cretaceous–Paleogene event; therefore, an equal number of lineages could have become extinct. All extant lineages of *Eucecidoses* appear to have begun diversifying around 5 Mya.

Although the credibility intervals associated with diversification plot are broad, date estimated still fits within the general trend of major geological changes and climatic shifts.

Because there were no evident topographic barriers in the southern portion of the continent, the advance of an Atlantic transgression, the Salamanca Sea, covered a great part of the continent during this period; this event might have had played a role on the early timing of diversification of the group [68].

*Eucecidoses* first diverged into two main lineages ca. 38 Mya, during the middle Eocene. One of these lineages includes the current populations from the South America Transition Zone (Lineage 6) and the Chacoan dominion (Lineage 5), which are highly divergent. Their split coincides with the ‘Inca phase’ of the Andean uplift and is correlated with an intense marine regression [68]. The other major lineage that subsequently diverged into other populations from the Parana forest and Chacoan provinces began this process during the Miocene, ca.20 Mya, coinciding with three more marine transgressions known as the Paranaean Sea, which separated a few land areas and gave rise to flooded regions that later came to the surface as plains. These newly formed ecosystems, combined with the tectonic “Quechua phase” of the Andes, created a rain shadow effect that led to the first stages of differentiation of the current biogeography of South America. Furthermore, during this same period of time and climatic conditions xeric environments became established in the south, and many plant families became more abundant, including the Anacardiaceae [68]. Although not the focus of the present study, altitude may play an important role in the distribution of *E*. *minutanus*, since *S*. *polygamus* populations are located at higher elevations. Plants located on hilltops are mainly used as hosts in the Pampean province (Southernmost Brazil, Uruguay and Buenos Aires region).

During the Quaternary, climate changes in the Neotropics contributed significantly to the diversification of fauna and flora [69]. Climatic oscillations and the glaciations may have caused the fragmentation of many habitats, creating temporary and isolated areas that resulted in refuges for many groups of species [70–72]. By detecting a possible origin of the *Eucecidoses* stock lineage in the south range in the Argentinian Monte we also inferred the existence of further recent northern diversification within both derived clades. This diversification is possibly associated with vicariant and dispersion events related either to forest refuge [73] or the emerged continental shelf during the Last Glacial Maximum (around 21 kyr), which allowed forests and forest-adapted species to expand [74]. We considered the scenario of recurrent dispersal from the same source population in the case of lineages 2, 3 and 4, which likely originated from the Pampean province and lately occupied eastern forests. This hypothesis is reinforced by the result of reciprocally monophyletic populations. The current distribution of lineage 2, which follows the same biogeographic province as some other populations of the Parana dominion, is clearly genetically isolated.

In vicariance, interacting lineages usually present the same distribution range, and consequently experience similar geological events that could lead to allopatric speciation, hence showing a concordant biogeographic pattern. The Cenozoic Andean uplift had a major impact on the evolution of South American landscapes, and was a fundamental event in generating the high biodiversity of the continent, providing ecological and vicariant events of speciation [75, 76]. Quaternary climatic fluctuations may also be indicative of vicariance that took place during the *Eucecidoses* speciation, as demonstrated for several other species [77–81].

### Host plant as driver of diversification

Geographically structured populations combined with genetic drift may be also going through a process of co-evolution (e.g., related to its host-plant), ultimately leading to isolation and speciation [77]. *Eucecidoses* date to the Early Cretaceous and *Schinus* the middle Miocene [82], which reveal a discrepancy in timing for co-diversification. Thus, we suggest that *Eucecidoses* may have originated first in distinct Anacardiaceae host and secondarily used *Schinus*. The significant increase in diversification rate of *Eucecidoses* around 5 Mya, is concordant with the rise of *Schinus*. Thus, the late diversification of *Eucecidoses* did not imply that they colonized already diversified *Schinus* host. Current knowledge of the *Eucecidoses-Schinus* interaction, based on insect-host ranges and their intimate relationship, suggest that ecological speciation may also have been driving species shifts in this case.

As already mentioned, specimens of *Schinus* used as hosts by *E*. *minutanus* have been identified in many herbaria as *Schinus polygamus* (Cav.) Cabrera (*sensu* Cabrera [28]; Fleig [29, 30]). The identity of these plants is however controversial, and the genus need to be reviewed (for a discussion, see [29–31, 83]). We have strong evidence from an ongoing study (Luz C.L.S., USP, unpublished data) that this taxon may be divided into several species in the near future. Consequently, *Schinus* specimens from Mendoza, Buenos Aires, Tucumán and Curitiba used in this study may in fact correspond respectively to *S*. *fasciculatus* (Griseb.) I.M. Johnst., *S*. *longifolius* (Lindl.) Speg., *S*. *gracilipes* I.M. Johnst. and *S*. *engleri* Barkley. In a follow-up study we will be looking for correspondence between times of divergence between hosts (*Schinus*) and gall inducers (*Eucecidoses*), as well as regarding their cladogenesis in a co-speciation scenario, taking advantage of a more accurate age recently proposed for Anacardiaceae, particularly *Schinus* [82]. Finally, to make robust inferences on host specialization more than co-phylogeny analysis might be required. An experiment involving phenotypic plasticity and relative fitness of *Eucecidoses* populations on local vs. non-local *Schinus* populations using transplants would be also important to clarify further the role of host plants in relation to evolution of the micromoths.

In summary, we clearly demonstrated in this study that variation in geographic distribution of this cecidosid moth in South America cannot be explained only by that of its host plant, which is much broader. The distribution pattern of *E*. *minutanus* coincides with the Peripampasic orogenic arc, with most populations occurring in the mountainous areas located east of the Andes (Argentinean Monte biogeographic province and Chacoan dominian/province) and on the Atlantic coast (Brazilian Parana and Araucaria forest provinces). Phylogeny and dating of clades based on molecular data performed with populations covering these areas corroborated this scenario from a historical perspective. Two *E*. *minutanus* clades began to split early (ca. 38 Mya) in association with these mountainous areas, and a few lineages differentiated further within each of these clades later in time (ca. 20 Mya). Thus, we associated the initial cladogenesis (first major clade) of *E*. *minutanus* with the Tertiary tectonics occurring in the area, starting with the uplift of the Andes. The second major clade, which includes lineage 1 (type locality) and lineages 2, 3 and 4, likely originated from recurrent dispersal of a meta-population from the central area north to the Pampean and further to coastal forests. The demographic dynamics of the six lineages across biogeographic regions was markedly different and the isolation by distance found among lineages reveals instability during glacial and interglacial periods. In this case study we clarified the evolutionary pathway from a phylogenetic perspective that this gall-inducing moth may have gone through in association with orogenic events that molded the mountainous regions located in the west and east South American coasts. Thus, our findings can be used to explain not only the evolutionary history of *E*. *minutanus*, but generally for regional Neotropical fauna.

## Supporting information

S1 Fig A, Dried-preserved *Schinus polygamus* branches from the Herbário do Instituto de Ciências Naturais, Universidade Federal do Rio Grande do Sul, Porto Alegre, Rio Grande do Sul, Brasil (ICN 043172), bearing open galls of *Cecidoses eremita* (indicated by open arrow) and *Eucecidoses minutanus* (closed arrows), shown in detail with their detached opercula in B and C, respectively. Scale bars = 3 and 2 mm, respectively.
(TIF)

S2 Fig Comparative assessment of BBM and S-DIVA analyses for ancestral area reconstruction in *Eucecidoses minutanus*. (TIF)

S1 Appendix Geographic data of *Schinus polygamus* (sensu lato) and *Eucecidoses minutanus* compiled for the biogeographic analysis. (PDF)

S1 Table Primers and PCR conditions to amplify the three mitochondrial loci (CoI, CoII and 16s) surveyed this study. (DOCX)

S2 Table Genbank accession numbers for individuals analysed in this study. (XLSX)

## References

[1] Rull V. Speciation timing and Neotropical biodiversity: the Tertiary–Quaternary debate in the light of molecular phylogenetic evidence. Mol Ecol. 2008; 17: 2722–2729. 10.1111/j.1365-294X.2008.03789.x 18494610

[2] Morrone JJ. Cladistic biogeography of the Neotropical region: identifying the main events in the diversification of the terrestrial biota. Cladistics. 2014b; 30: 202–214. 10.1111/cla.12039 34784690

[3] Cox CB, Moore PD. Biogeography: An Ecological and Evolutionary Approach. 8th ed Chichester, UK: John Wiley & Sons; 2010. 440p.

[4] Matos-Maraví PF, Peña C, Willmott KR, Freitas AVL, Walhlberg N. Systematics and evolutionary history of butterflies in the ‘‘Taygetis clade” (Nymphalidae: Satyrinae: Euptychiina): Towards a better understanding of Neotropical biogeography. Mol Phylogenet Evol. 2012; 66: 54–68. 10.1016/j.ympev.2012.09.005 23000820

[5] Turchetto-Zolet AC, Pinheiro F, Salgueiro F, Palma-Silva C. Phylogeographical patterns shed light on evolutionary process in South America. Mol Ecol. 2013; 22: 1193–1213. 10.1111/mec.12164 23279129

[6] Daza JM, Smith EN, Páez VP, Parkinson CL. Complex evolution in the Neotropics: The origin and diversification of the widespread genus *Leptodeira*(Serpentes: Colubridae). Mol Phyl Evol. 2009; 53: 653–67. 10.1016/j.ympev.2009.07.022 19643196

[7] Morrone JJ. La zona de transiciónsudamaericana: Caracterización y relevância evolutiva. Acta Entomol Chilena. 2004; 28: 41–50.

[8] Ringuelet RA. Rasgos fundamentales de lazoogeografia Argentina. Physis. 1961; 22: 151–170.

[9] Mattoni CI, Acosta LE. Scorpions of the insular Sierras in the Llanos District (province de La Rioja, Argentina) and theirzoogeographical links. Biogeographica. 1997; 73: 67–80.

[10] Crisci JV, Freire SE, Sancho G, Katinas L. Historical biogeography of the Asteraceae from Tandilia and Ventania mountain ranges (Buenos Aires, Argentina). Caldasia. 2001; 23: 21–41.

[11] Ferretti N, González A, Pérez-Miles F. Historical biogeography of mygalomorph spiders from the peripampasic orogenic arc based on track analysis and PAE as a panbiogeographical tool. Syst Biodivers. 2012: 10: 179–193.

[12] Acosta LE. Escorpiones y opiliones de laprovincia de Córdoba: diversidad y zoogeografía. Bull Soc Neuchâtel Sci Nat. 1991; 116: 11–17.

[13] Pérez-Losada M, Bond-Buckup G, Jara CG, Crandall KA. Molecular systematics and biogeography of the Southern South American freshwater “Crabs” Aegla (Decapoda: Anomura: Aeglidae) using multiple heuristic tree search approaches. Syst Biol. 2004; 53: 767–780. 10.1080/10635150490522331 15545254

[14] Davis DR. The Monotrysian Heteroneura In: Kristensen NPeditor. Handbook of Zoology, Lepidoptera, Moths and Butterflies, vol 1: Evolution, Systematics, and Biogeography. Berlin & New York: Walter de Gruyter; 1998 pp. 65–90.

[15] Pellmyr O, Leebens-Mack J. Forty million years of mutualism: evidence for Eocene origin of the yucca-yucca moth association. Proc Natl AcadSci USA. 1999; 96: 9178–9183. 10.1073/pnas.96.16.9178 PMC17753 10430916

[16] Hoare RJB, Dugdale JS. Description of the New Zealand incurvarioid *Xanadoses nielseni*, gen. nov., sp. nov. and placement in Cecidosidae (Lepidoptera). Invertebr Syst. 2003; 17: 47–57.

[17] Moreira GRP, Eltz RP, Pase RB, Silva GT, Bordignon SAL, Mey W, Gonçalves GL. *Cecidonius pampeanus*, gen. et sp. n.: an overlooked and rare, new gall-inducing micromoth associated with *Schinus* in southern Brazil (Lepidoptera, Cecidosidae). ZooKeys. 2017; 695: 37–74. 10.3897/zookeys.695.13320 PMC5673834 29134006

[18] Morrone JJ. Biogeographical regionalization of the Neotropical region. Zootaxa. 2014a; 3782: 1–110. 10.11646/zootaxa.3782.1.1 24871951

[19] Carnaval AC, Moritz C. Historical climate modeling predicts patterns of current biodiversity in the Brazilian Atlantic forest. J Biogeogr. 2008; 35: 1187–1201.

[20] Carnaval AC, Hickerson MJ, Haddad CFB, Rodrigues MT, Moritz C. Stability predicts genetic diversity in the Brazilian Atlantic forest hotspot. Science. 2009; 23: 785–789. 10.1126/science.1166955 19197066

[21] Maestri R, Fornel R, Gonçalves GL, Geise L, Freitas TRO, Carnaval AC. Predictors of intraspecific morphological variability in a tropical hotspot: comparing the influence of random and non-random factors. J Biogeogr. 2016; 43: 2160–2172.

[22] Brèthes J. Estudio fito-zoológico sobre algunos lepidópteros argentinos productores de agallas. AnalesSoc Ci Argent. 1916; 82: 113–140.

[23] Moreira GRP, Gonçalves GL, Eltz RP, San Blas G, Davis DR. Revalidation of *Oliera* Brèthes (Lepidoptera: Cecidosidae) based on a redescription of *O*. *argentinana* and DNA analysis of Neotropical cecidosids. Zootaxa. 2012; 3557: 1–19.

[24] Loetti V, Valverde A, Rubel DN. Gallsof Cecidoses eremita Curtis and *Eucecidoses minutanus* Brèthes (Lepidoptera: Cecidosidae) in Magdalena, Buenos Aires Province: preliminarystudyandassociated fauna. Biota Neotrop 2016; 16 (4): e20160161.

[25] Becker VO. The taxonomic position of the Cecidosidae Brèthes (Lepidoptera). Pol Pis Entomol. 1977; 47: 79–86.

[26] Curtis J. On a species of moth found inhabiting the galls of a plant near to Monte Video. Trans ZoolSoc London. 1835; 3: 19–20.

[27] Steibel PE, Troiani HO. La identidad de *Schinus fasciculatus* var. *arenicola* y rehabilitación de *S*. *sinuatus* (Anacardiaceae). B SocArgent Bot. 2008; 43: 15–166.

[28] Cabrera AL. Revisión de las Anacardiáceas Austroamericanas. Rev Mus La Plata. 1938; 2: 3–64.

[29] Fleig M. Anacardiaceae Boletim Instituto de Biociências. Flora Ilustrada do Rio Grande do Sul, 18. 1987; 42: 1–72.

[30] Fleig M. Anacardiáceas In: Reitz R editor. Flora Ilustrada Catarinense, parte I. Itajaí: Herbário Barbosa Rodrigues: Empasc; 1989 pp. 1–64.

[31] Barkley FA. A study of *Schinus* L. Lilloa. 1957; 28: 1–110.

[32] Luz CLS.Anacardiaceae R. Br. na Flora Fanerogâmica do Estado de São Paulo. M.Sc. Thesis, Universidade de São Paulo, Instituto de Biociências. 2011. Available: http://www.teses.usp.br/teses/disponiveis/41/41132/tde-27042012-123436/pt-br.php

[33] Caterino MS, Sperling FAH. *Papilio* Phylogeny based on mitochondrial Cytochrome Oxidase I and II Genes. Mol Phylogenet Evol. 1999; 11 (1): 122–137. 10.1006/mpev.1998.0549 10082616

[34] Palumbi SR. PCR and molecular systematics In Molecular Systematics, 2nd edition, Hillis D., Moritz C., and Mable B., Eds. Sinauer Press 1996.

[35] Lanfear R, Calcott B, Ho SYW, Guindon S. PartitionFinder: combined selection of portioning schemes and substitution models for phylogenetic analyses. Mol Biol Evol. 2012; 29 (6): 1695–1701. 10.1093/molbev/mss020 22319168

[36] Guindon S, Gascuel O. A simple, fast, and accurate algorithm to estimate large phylogenies by maximum likelihood. Syst. Biol. 2003; 52 (5): 696–704. 14530136 10.1080/10635150390235520

[37] Hillis DM, Bull JJ. An empirical test of bootstrapping as a method for assessing confidence in phylogenetic analysis. Syst. Biol. 1993; 42:182–192.

[38] Drummond AJ, Suchard MA, Xie D, Rambaut A. Bayesian Phylogenetics with BEAUti and BEAST 1.7. Mol Bio lEvol. 2012; 29 (8): 1969–1973. 10.1093/molbev/mss075 PMC3408070 22367748

[39] Bouckaert RR. DensiTree: making sense of sets of phylogenetic trees. Bioinformatics. 2010; 26 (10): 1372–1373. 10.1093/bioinformatics/btq110 20228129

[40] Erixon P, Svennblad B, Britton T, Oxelman B. Reliability of Bayesian Posterior Probabilities and Bootstrap Frequencies in Phylogenetics. Syst. Biol. 2003; 52 (5): 665–673. 14530133 10.1080/10635150390235485

[41] Nielsen ES, Davis DR. The first southern hemisphere prodoxid and the phylogeny of the Incurvarioidea (Lepidoptera).Syst Entomol. 1985; 10: 307–322.

[42] Regier JC, Mitter C, Kristensen NP, Davis DR, van Nieukerken EJ, Rota J, Simonsen TJ, Mitter KT, et al A molecular phylogeny for the oldest (nonditrysian) lineages of extant Lepidoptera, with implications for classification, comparative morphology and life-history evolution. Syst Entomol. 2015; 40 (4): 671–704.

[43] Drummond AJ, Ho SYW, Phillips MJ, Rambaut A. Relaxed phylogenetics and dating with confidence. PLoS Biol. 2006; 4 (5): e88 10.1371/journal.pbio.0040088 16683862 PMC1395354

[44] Ho SYW, Phillips MJ. Accounting for calibration uncertainty in phylogenetic estimation of evolutionary divergence times. Syst. Biol. 2009; 58(3): 367–380 10.1093/sysbio/syp035 20525591

[45] Walhberg N, Wheat CW, Peña C. Timing and Patterns in the Taxonomic Diversification of Lepidoptera (Butterflies and Moths). PLoS ONE. 2013; 8 (11): e80875 10.1371/journal.pone.0080875 24282557 PMC3839996

[46] Whalley PES New taxa of fossil and recent Micropterigidae with a discussion of their evolution and a comment on the evolution of Lepidoptera (Insecta) Ann. Transvaal Mus. 1978; 31: 71–86.

[47] Excoffier L, Lischer HEL. Arlequin suite ver 3.5: A new series of programs to perform population genetics analyses under Linux and Windows. Mol Ecol Resour. 2010; 10: 564–567. 10.1111/j.1755-0998.2010.02847.x 21565059

[48] Excoffier L, Smouse PE, Quattro JM. Analysis of molecular variance inferred from metric distances among DNA haplotypes: application to human mitochondrial DNA restriction data. Genetics. 1992; 131: 479–491. 1644282 10.1093/genetics/131.2.479 PMC1205020

[49] Kimura M. A simple method for estimating evolutionary rate of base substitutions through comparative studies of nucleotide sequences. J. Mol. Evol. 1980; 16: 111–120. 7463489 10.1007/BF01731581

[50] Bandelt HJ, Forster P, Röhl A. Median-joining networks for inferring intraspecific phylogenies. Mol Biol Evol. 1999; 16: 37–48. 10.1093/oxfordjournals.molbev.a026036 10331250

[51] Mantel N. The detection of disease clustering and a generalized regression approach. Cancer Res. 1967; 27: 209–220. 6018555

[52] Fu Y-X. Statistical tests of neutrality of mutations against Population Growth, Hitchhiking and background selection. Genetics. 1997; 147: 915–925. 9335623 10.1093/genetics/147.2.915 PMC1208208

[53] Harpending RC. Signature of ancient population growth in a low-resolution mitochondrial DNA mismatch distribution. Hum Biol.1994; 66: 591–600. 8088750

[54] Ronquist F.Dispersal-vicariance analysis: a new approach to the quantification of historical biogeography. Syst Biol. 1997; 46: 195–203.

[55] Yu Y, Harris AJ, He X. S-DIVA (Statistical Dispersal-Vicariance Analysis): a tool for inferring biogeographic histories. MolPhylEvol. 2010; 56: 848–850. 10.1016/j.ympev.2010.04.011 20399277

[56] Rambaut A, Drummond AJ. Tracer version 1.5 [computer program]. http://beast.bio.ed.ac.uk. 2009.

[57] Yu Y, Harris AJ, Blair C, He X. RASP (Reconstruct Ancestral State in Phylogenies): a tool for historical biogeography. MolPhylEvol. 2015; 87: 46–49. 10.1016/j.ympev.2015.03.008 25819445

[58] Pybus OG, Harvey PH. Testing macro-evolutionary models using incomplete molecular phylogenies. Proc. R. Soc. Lond. B. 2000; 267: 2267–2272. 10.1098/rspb.2000.1278 PMC1690817 11413642

[59] Malcolm M, Oggero AJ, Arana MD, Tordable MC, Boito GR. Los insectosgalícolasen Schinus fasciculata (Anacardiaceae) enel Espinaldel centro de Argentina. Iheringia Ser Zool. 2015; 105: 133–139.

[60] Fernández S, D´Ambrogio A. Cecidiosen uma Anacardiácea patagônica. B Soc Argent Bot. 2001: 36: 243–251.

[61] Kirichenko N, Huemer P, Deutsch H, Triberti P, Rougerie R, Lopez-Vaamonde C. Integrative taxonomy reveals a new species of *Callisto* (Lepidoptera, Gracillariidae) in the Alps. ZooKeys. 2015; 473: 157–176. 10.3897/zookeys.473.8543 PMC4304045 25632257

[62] Zera AJ, Denno R F. Phisiology and Ecology of dispersal polymorphism in insects. Annu Rev Entomol. 1997; 42: 207–230. 10.1146/annurev.ento.42.1.207 15012313

[63] Wille J. *Cecidoses eremita* Curt. und ihregalle an *Schinus dependens* Ortega. Zoomorphology. 1926; 7: 1–101.

[64] San Blas G, Davis D. Redescription of Dicranoses capsulifex Kieffer and Jörgensen (Lepidoptera: Cecidosidae) with description of the immature stages and biology. Zootaxa. 2013; 3682: 371–384. 25243292 10.11646/zootaxa.3682.2.9

[65] Brower AVZ. Rapid morphological radiation and convergence among races of the butterfly *Heliconius erato* inferred from patterns of mitochondrial DNA Evolution. Proc Natl Acad Sci U S A. 1994; 91:6491–6495. 8022810 10.1073/pnas.91.14.6491 PMC44228

[66] Cherry JL. Should We Expect Substitution Rate to Depend on Population Size? Genetics. 1998; 150: 911–919. 9755219 10.1093/genetics/150.2.911 PMC1460373

[67] Felsenstein J. Accuracy of coalescent likelihood estimates: do we need more sites, more sequences, or more loci? Mol Biol Evol. 2006; 23: 691–700. 10.1093/molbev/msj079 16364968

[68] Ortiz-Jaureguizar E, Cladera G. Paleontoenvironmental evolution of southern South America during the Cenozoic. J Arid Environ. 2006; 66: 498–532.

[69] Rull V. Quaternary speciation in the Neotropics. Mol Ecol. 2006; 15: 4257–4259. 10.1111/j.1365-294X.2006.03052.x 17054517

[70] Antonelli A, Sanmartín I. Why are there so many plant species in the Neotropics? Taxon. 2011; 60 (2): 403–414.

[71] Chaves JA, Weir JT, Smith TB. Diversification in Adelomyia hummingbirds follows Andean uplift. Mol Ecol. 2011: 20 (21): 4564–4576. 10.1111/j.1365-294X.2011.05304.x 21981387

[72] Maia AVP, Almeida C, Santoro KR, Melo JLA, Oliveira JV, Guedes RNC, Badji CA. High-level phylogeographic structuring of Neoleucinodeselegantalis Guenée (Lepidoptera, Crambidae) in Brazil: an important tomato pest. Rev Bras Entomol. 2016; 60: 206–210.

[73] Peçanha WT, Althoff SL, Galiano D, Quintela FM, Maestri R, Gonçalves GL, et al Pleistocene climatic oscillations in Neotropical open areas: Refuge isolation in the rodent *Oxymycterus nasutus* endemic to grasslands. PLoS ONE; 2017: 12(11): e0187329 10.1371/journal.pone.0187329 29176839 PMC5703582

[74] Leite YL, Costa LP, Loss AC, Rocha RG, Batalha-Filho H, Bastos AC, et al Neotropical forest expansion during the last glacial period challenges refuge hypothesis. Proc Natl AcadSci U S A. 2016; 113:1008–13. 10.1073/pnas.1513062113 PMC4743791 26755597

[75] Elias M, Joron M, Wllmott K, Silva-Brandão KL, Kaise V, Arias CF, et al Out of the Andes: patterns of diversification in clearwing butterflies. Mol Ecol. 2009; 18: 1716–1729. 10.1111/j.1365-294X.2009.04149.x 19386035

[76] Hoorn C, Wesselingh FP, terSteege H, Bermudez MA, Mora A, Sevink J, et al Amazonia Through Time: Andean Uplift, Climate Change, Landscape Evolution, and Biodiversity. Science. 2010; 330 (2006): 927–931. 21071659 10.1126/science.1194585

[77] Althoff DM, Segraves KA, Smith CI, Leebens-Mack J, Pellmyr O. Geographic isolation trumps coevolution as a driver of yucca and yucca moth diversification. Mol Phylogenet Evol. 2012; 62: 898–906. 10.1016/j.ympev.2011.11.024 22178365

[78] Brunes TO, Thomé MTC, Alexandrino J, Haddad CFB, Sequeira F. Ancient divergence and recent population expansion in a leaf frog endemic to the southern Brazilian Atlantic forest. Org Divers Evol. 2015; 15: 595–710.

[79] Hall JPW. Montane speciation patterns in Ithomiola butterflies (Lepidoptera: Riodinidae): are they consistently moving up in the world? P R Soc B. 2005; 272: 2457–2466. 10.1098/rspb.2005.3254 PMC1599773 16271969

[80] Hewitt G. The genetic legacy of the Quaternary ice ages. Nature. 2000; 405: 907–913. 10.1038/35016000 10879524

[81] Paula AS, Diotaiuti L, Galvão C. Systematics and biogeography of Rhodniini (Heteroptera: Reduviidae: Triatominae) based on 16S mitochondrial rDNA sequences. J Biogeogr. 2007; 34: 699–712.

[82] Muellner-Riehl AN, Weeks A, Clayton JW, Buerki S, Nauheimer L, Chiang Y-C; Cody S, Pell SK. Molecular phylogenetics and molecular clock dating of Sapindales based on plastid rbcL, atpB and trnL-trnF DNA sequences. Taxon. 2016; 65: 1019–1036

[83] Burckhardt D, Basset Y. The jumping plant-lice (Hemiptera, Psylloidea) associated with *Schinus* (Anacardiaceae): systematics, biogeography and host plant relationships. J Nat Hist. 2000; 34: 57–155.

