# Supplementary figures and images for: Phylogeography of the gall-inducing micromoth Eucecidoses minutanus Brèthes (Cecidosidae) reveals lineage diversification associated with the Neotropical Peripampasic Orogenic Arc
Source: PLoS One. 2018 Aug 8;13(8):e0201251. doi: 10.1371/journal.pone.0201251 (PMC6082564; doi:10.1371/journal.pone.0201251)

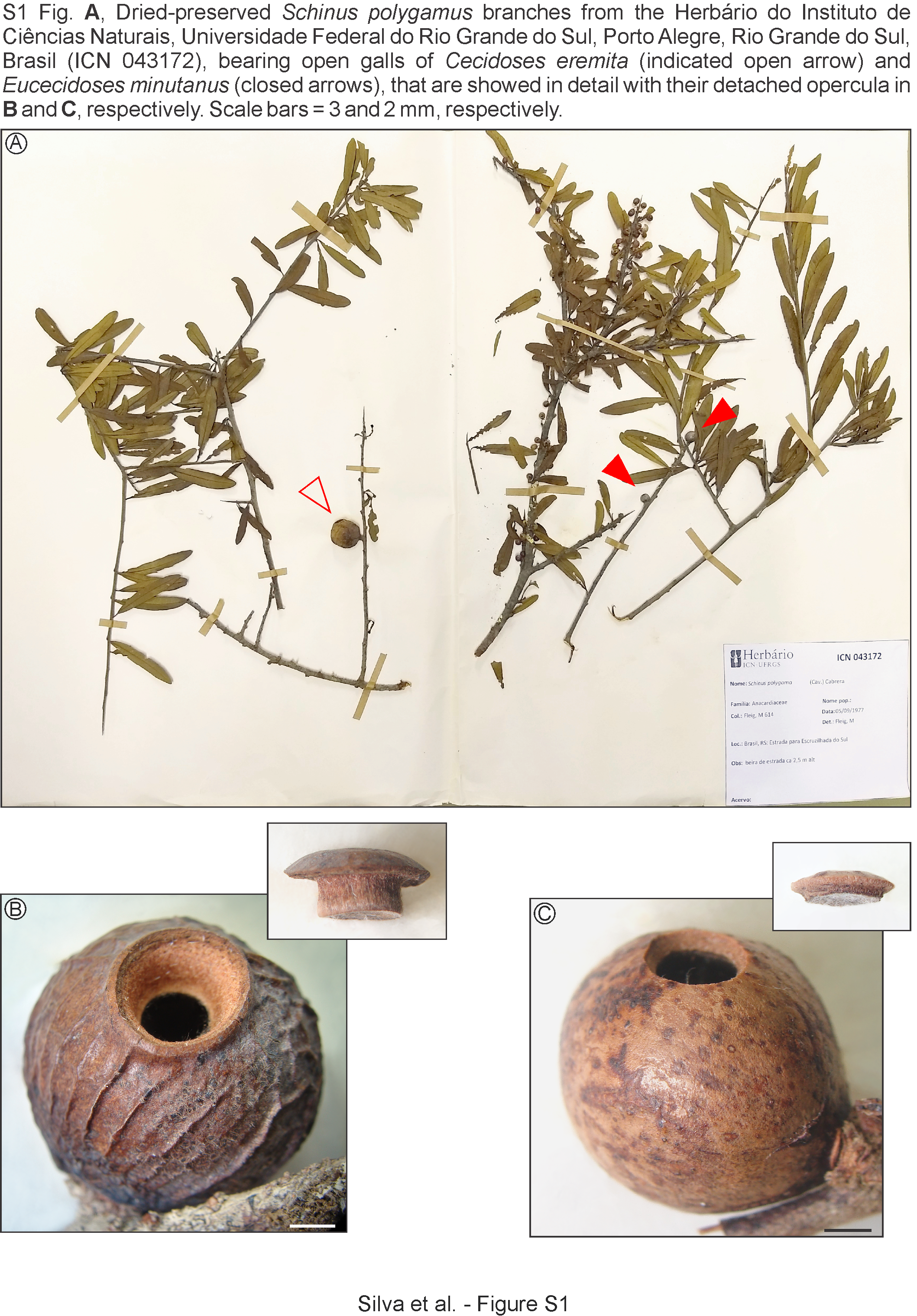

Supplement: S1 Fig — A, Dried-preserved Schinus polygamus branches from the Herbário do Instituto de Ciências Naturais, Universidade Federal do Rio Grande do Sul, Porto Alegre, Rio Grande do Sul, Brasil (ICN 043172), bearing open galls of Cecidoses eremita (indicated by open arrow) and Eucecidoses minutanus (closed arrows), shown in detail with their detached opercula in B and C, respectively. Scale bars = 3 and 2 mm, respectively. (TIF) [file pone.0201251.s001.tif]

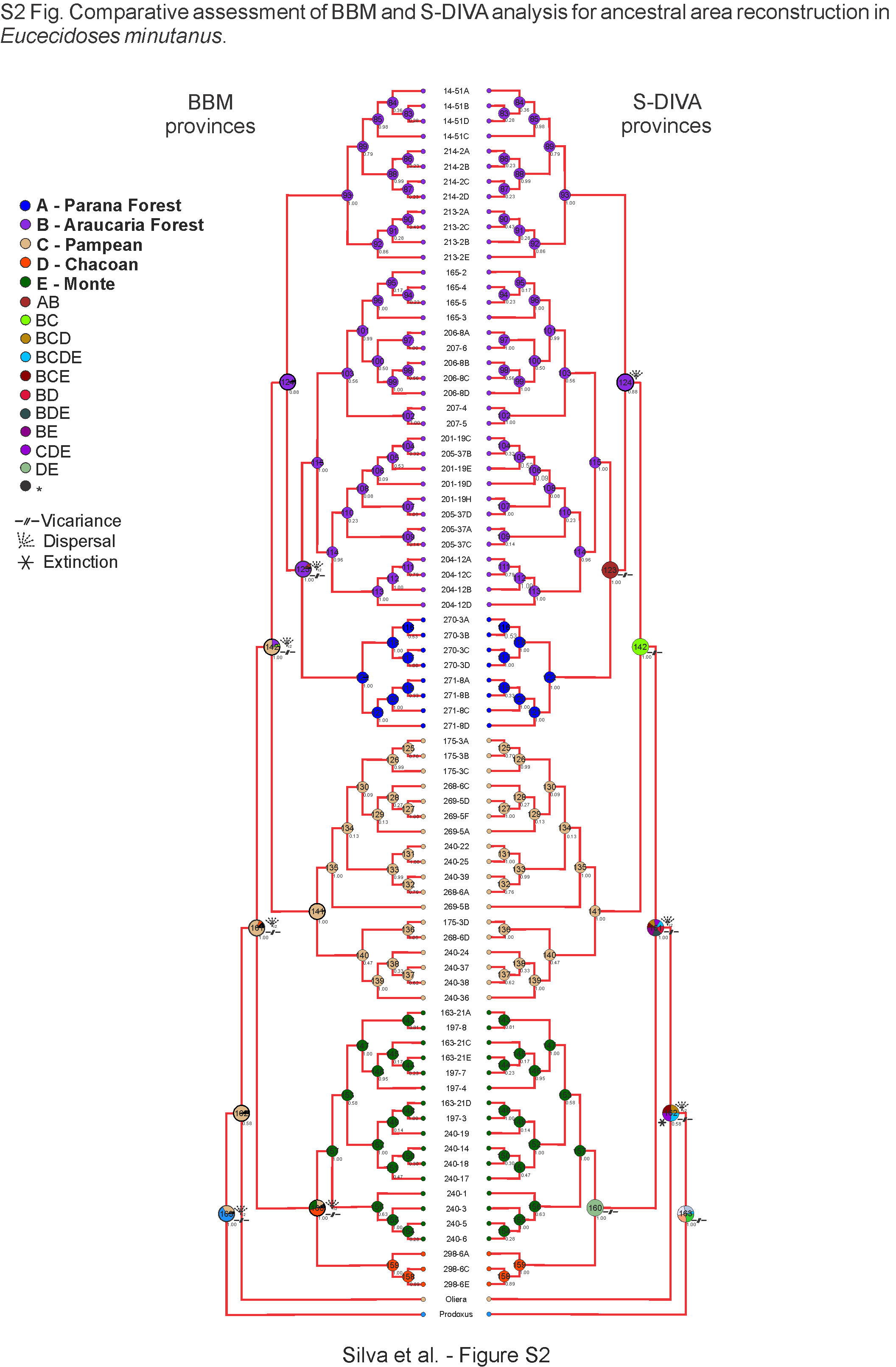

Supplement: S2 Fig — (TIF) [file pone.0201251.s002.tif]
