# Supplementary material for: Phylogeography of the gall-inducing micromoth Eucecidoses minutanus Brèthes (Cecidosidae) reveals lineage diversification associated with the Neotropical Peripampasic Orogenic Arc
Source: PLoS One. 2018 Aug 8;13(8):e0201251. doi: 10.1371/journal.pone.0201251 (PMC6082564; doi:10.1371/journal.pone.0201251)
Supplement: S1 Appendix — (PDF) [file pone.0201251.s003.pdf]

S1 Appendix. Geographical data of *Schinus polygamus* (lato sensu) and *Eucecidoses minutanus* compiled for the biogeographical analysis. Data from literature are followed by the corresponding references within brackets, as follows: 1 – SpeciesLink (splink.cria.org.br); 2 - Fleig (1989); 3- Barkley, F. A. (1944); 4 – German San Blas, UNLP, inedit data; 5 – Loetti et al. (2016); 6 – Plants Jstor (plants.jstor.org); 7 – Museo de La Plata; 8 – Intituto de Botánica Darwinion; 9 – Museo Argentino de Ciencias Naturales Bernardino Rivadavia; 10 – Facultad de Agronomia de Montevideo; 11 – Gilson R.P. Moreira, UFRGS, inedit data (dried material preserved at Laboratório de Morfologia e Comportamento de Insetos (LMCI); to be donated to ICN). a – as *S. longifolius*; b – as *S. spinosus*; c – as *S. dependens*; d – as *S. johnstonii*; e – as *S. polygama*; f – as *S. sinuatus*; g – as *S. piliferus*; h – as *S. fasciculatus*; i – as *S. cabrerae*; j – as *S. engleri*; k – as *S. apparicianus*; l – as *S. ferox*; m – as *S. praecox*; n – as *S. bumelioides*; * indicates records of *E. minutanus* galls. Abbreviations follow Thiers, B. [continuously updated], except for BCTw (Xiloteca Calvino Mainieri, Instituto de Pesquisas Tecnológicas, São Paulo, Brazil), DVPR (Herbário da Universidade Tecnológica do Paraná, Dois Vizinhos, Paraná, Brasil), FPS (Fototeca Paulo Schwirkowski, São Bento do Sul, Santa Catarina, Brasil). Latitude (S) and longitude (W) are given at the end in parentheses.

Schinus polygamus: ARGENTINA: **Buenos Aires:** *Atalaya* – [9], Izunieta, BA68381, 20/03/1970, (-35.0333, -57.5333); *Atucha* – [1], Krapovickas A 3283, SP 54306, 26/01/1947, (-33.9736, -59.2025); *Bahia Blanca* – [3,d], Eyerdam, Beetle & Grondona 23449, GH, 16/12/1938, (-38.7166, -62.2666); *Berisso* – [3,a], Cabrera 507, GH, 11/1928, (-34.8666, -57.8666); *Brandsen* – [4*], GSB, R. & E. San Blas, C. Amaya, 06/09/2012 (-35.1750, -58.2365); *Bernal* – [7,a], Elián L. Guerrero, LP 114, 28/08/2011,(-34.7, -58.2833); *Coronel Rosales* – [6,d], Boelcke, O.11936, 08 /12/1964, Múlgura de Romero, M. E., (-38.8666, -62.0666); *Estancia José Santos Arevalo* – [7], L. R. Miccio, LP 795, 14/11,(-35.1667, -59.1667); *Estanción San Isidro* – [7,a], Torres Robles, LP 342, 06/12/2000, (-35.1458, -57.3738); *Isla Martin Garcia* – [7,a*], Torres Robles, P. Simon, N. González, LP 2022, 30/10/2004, (-34.1798, -58.25); *Laguna Chasilauquen* – [7], A. L. Cabrera, H. A. Fabris, LP 14809, 17/11/1962, (-37.2727, -63.1536); *La Plata* – [6,a], SI045512, 17/10/1929, Muñoz, J. D. det., (-34.9213, -57.9544); *Las Palmas* – [6,a*], Boelcke, O. 4951, SI045514, 18/10/1951, Muñoz, J. D. det., (-34.0194, -59.1341); *Lima* – [8,h], Múlgura de Romero et al., SI 324, 08/12/1982, (-34.033333, -59.2); *Lujan* – [2], F. A. Barkley 8227, LIL, 26/09/1942, (-34.5666, -59.1); *Magdalena* – [5,a*], 04/2013, (-35.0833, -57.5166); *Monte Hermoso* – [7,d], H. A. Fabris, H. Schwabe, LP 4788, 24/11/1962, (-38.9833, -61.3); *Punta Lara* – [3,a*], 1815, GH, 25/10/1931, (-34.8166, -57.9666); *Quilmes* – [7,a], Nieves Baldaccini, LP 32, 16/11/2014, (-34.7333, -58.2666); *San Isidro* – [6,a*], Hicken, C. M., SI045513, 12/12/1901, Muñoz, J. D.det., (-34.4666, -58.5166); *Sierra de las Tunas* – [7,h], Laura A. Pertusi, LP 52, 25/11/1979, (-38.9494, -61.8155); *Tigre* – [6,a], Lanfranchi, A. E. 1685, SI045506, 15/09/1967, (-34.4258, -58.5966); *Tornquist*– [7,h], Proyeto Ventania, LP 233, 26/11/1978, Laura Pertusi, det., (-38.1, -62.2333); *Zarate* – [4*], GSB, R. y E. San Blas, C. Amaya, 08/09/2012 (-34.0952, -59.0241); **Catamarca:** *Aldalgalá* – [2], Vervoort 3428, LIL, (-27.6, -66.3166); *Belén* – [7*], A.L. Cabrera, E. M. Zardini, N. Deginani, LP 24654, 27/01/1974, (-27.65, - 67.0333); *Capayan* – [2], Risso 870, LIL, (-28.7636, -66.0683); *El Alto* – [6,a], Venturi, S. 7063, SI033348, 10/01/1928, Múlgura de Romero, M. E. det., (-28.3, -65.3666); *El Durazno* – [8,h*], A. R. Cuezzo 9494C., SI, 14/11/1972, (-27.2538, -67.0380); *La Merced* – [4*], GSB, 09/07/2014, 27/08/2014, 23/11/2015 (28.1644, -65.6516); *La Puerta* – [7], A. L. Cabrera, LP 9523, 17/08/1969, (-28.1666, -65.7833); *San Antonio* – [8*], Sara T., SI, 25/01/1957, (-28.0333, -65.6833); *Shincal* – [7,h], Capparelli Aylen, LP 31, 23/05/1991, Laura Iarleghi det., (-27.6861, -67.1805); **Chaco:** *Barranqueras* – [3,f], Curran 15, US, 12/11/1913, (-27.4833, -58.9333); *Chacabuco* – [1,h], Scarpa, G 797, SPF 214453, 04/03/2009, Scarpa, G det., (-27.2166, -61.2); *Colonia Benitez* – [3,f], Schulz 848, 03/1935, (-27.3333, -58.9333); *Las Breñas* – [8,h], Aliscioni, S.S. et al., SI 663, 21/03/2006, (-27.0666, -61.0666); *La Fidelidad* – [3,f], Jorgensen 1950, FAB/NY, 09/04/1918, (-24.9677, -60.9666); *Resistencia* – [2], Margarita Belen, LIL 201713, 13/03/1947, (-27.4513, -58.9866); *Villa Angela* – [3,f], Bogga 8, NY, 03/01/1930, (-27.5833, -60.7166); **Chubut:** *Colhue Hualpi* – [8,d], Martin T., SI 11, 26/12/1972, (-45.4938, -68.7572); *Futaleufú* – [6,d], Illin, N. 82, SI033252, (-42.9, -71.3166); *Gaiman* – [1,d], Seijo, G 1509, MBM 242133,

20/01/1999, Schinini, A det., (-43.2833, -65.4833); *Istmo Carlos Ameghino*– [7,d], Juan Daciuk, LP 126, 3/10/1969, Juan Daciuk det., (-42.4526, -64.5); *Nueva Lubecka* – [8,h], Zuloaga, F. O., SI 13926, 19/11/2012, Rau det., (-44.5333, -70.4)*; Paso de Indios* – [6,d], Biganzoli, F.; Larsen, C. 1930, SI033248, 01/11/2008, Múlgura de Romero, M. E. det., (-43.9, -69.0666); *Península Valdés* – [7,d], Juan Daciuk, LP 47, 15/08/1969, Juan Daciuk det., (-42.5, -63.9333); *Pico Salamanca* – [7], Castellanos, LP 6086, 25/01/1932,(-45.5733, -67.3372); *Puerto Madryn* – [8,d*], L. Hauman, SI 14, 07/01/1914, F. A. Barkley det., (-42.8295, -65.0823); *Puerto Pirámides* – [3,d], Eyerdam, Beetle & Grondona 23570, GH, 21/12/1938, (-42.5666, -64.2833); *Punta Gales* – [6,d], Daciuk, J. 21, SI033249, 11/09/1969, Múlgura de Romero, M. E. det., (-42.4236, -5358); *Punta Loma* – [9,d*], J. Daciuk, BA 9824, 11/01/1968, M. J. Dimitri det., (-42.8166, -64.8833); *Rawson* – [7*], M. M. Job, LP 3011, 20,11/1951, (-43.3, -65.1); *Sarmiento* – [6,d], Kreibohm, E. 273, SI033259, 31/10/1965, Terrazas, T. det., (-45.6, -69.0833); **Cordoba:** *Capilla del Monte* – [7,h], Maria M. Job, LP, 18/08/1952, (-30.85, -64.5166); *Córdoba* – [7,h], J. H. Hunziker, LP 2654, 01/1950, (-31.4166, -64.1833)*; El Brete* – [3,f], Lorentz 1477, F, 02/02/1878, (-30.6905, -64.8747); *La Cumbre* – [6,h], Cuezzo, A.R & Balegno, B. 2128, E00089920, 10/02/1950, (-31.1, -64.483); *La Falda* – [3,h], Job 545, NY, 01/1936, (-31.0833, -64.5); *Malagueño* – [8], A. M. Fuchs, SI 17099, 12/08/1949, (-31.4638, -64.3575); *Mina Clavero* – [8,h], Cordo, H., SI 77-A-56, 03/12/1976, (-31.7238, -65.005); *Nono* – [1,h], Silva-Luz, CL; Luz, LF 278, SPF 212447, 22/03/2014, Silva-Luz, CL det., (-31.8104, -64.9928); *Punilla* – [1,m], Hunziker, AT 7451, MBM 144399, 17/10/1947, F. A. Barkley, det., (-31.25, -64.45); *Rio Primero* – [3,a], Hyeronymus, F, 30/08/1877, (-31.1333, -63.3666); *San Javier* – [1,m], Castellanos, A 3, SPF 214454, 15/12/1927, Silva-Luz, CL det., (-31.9333, -65.2); *San Roque* – [3,d], Rose & Russel 21059, US, 09/09/1915, (-31.3730, -64.4416); *Sierra Achala* – [3,d], Hyeronymus, NY, 11/11/1878, (-31.6826, -64.8371); *Sierra de los Condores* – [8], Kiesling, R., Terrazas, T., SI 10115, 04/11/2004, M. E. Múlgura, det., (-32.3211, -64.28); *Tanti* – [1,m], Silva-Luz, CL; Luz, LF 280, SPF 212432, 23/03/2014, Silva-Luz, CL det., (-31.3333, -64.6); *Unquillo* – [3,f], Bruch 2935, NY, 1926, (-31.2333, -64.3166); *Villa La Merced* – [3,h], Hieronymus, NY, 11/1891, (-31.8238, -64.5194); **Corrientes:** *Arroyo Timboy* – [4*], GSB, 21/01/2012, (-30.0638, -57.8722); *Concepción* – [7,j], Troels Myndel Pedersen, LP 4632, 24/09/1974, (-28.3666, -57.8666); *Curuzú Cuatiá* – [8,h], A. Schinini, O Ahumada, SI 13917, 08/01/1977, (-29.7833, -58.0833); *Empedrado* – [7,h], Troels Myndel Pedersen, LP 1755, 18/06/1952, (-27.9333, -58.7833); *Garruchos* – [7,n], Krapovickas, Cristóbal, Tressens, Schinini, Quarin, CTES 25062, 12/04/1974, (-28.1736, -55.6511); *Gioya* – [3,f], Curran 335, US, 04/09/1913, (-29.1333, -59.25); *Itatí* – [7,h], A. Schinini, L. Mroginski, LP 4486, 16/04/1972, (-27.2666, -58.25); *Mburucuyá* – [7,n], Troels Myndel Pedersen, LP 4499, 16/03/1958, (-28.05, -58.2333); *Mercedes* – [1], Marchiori, J. N. C. s.n., HDCF 503, 26/08/1981, Longhi, S. det., (-29.1838, -58.0733); *San Cosme* – [1,e], Paula-Souza, J.; Ferrucci, M.S.; Meza Torres, E.I. 8060, ESA098907, 31/01/2007,(-27.5166, -58.5666); *San Luis del Palmar* – [1], Krapovickas, A; Quarín, C; Fernández, A 21836, MBM 23887, 08/03/1972, (-27.5108, -58.5569); **Entre Ríos:** *Concepcion del Uruguay* – [3,a], Lorentz, GH, 11/11/1877, (-32.4844, -58.2369); *El Espinillo* – [7,a], Muñoz, J.D., SI 611, 30/01/1980, Malúlgura det., (-33.525, -58.3555); *Gualeguaychú* – [2], Meyer 10276, LIL, (-33.0077, -58.5111); *Parana* – [1,f], Silva-Luz, CL 330, SPF 216474, 21/12/2014, Silva-Luz, CL det., (-31.7372, -60.3284); *Santa Elena* – [7,h], A. Burkart et al., SI 28736, 23/10/1971, (-30.9413, -59.7833); *Yuqueri* – [7,] Maria M. Job, 03/11/1949, (-31.3833, -58.1192); **Formosa:** *Espinillo* – [6,h], Morel, I. 7776, E00089918, 27/05/1949, F. A. Barkley det, (-24.9666, -58.5666)*; Formosa* – [3,f], Jorgensen 1971, FAB/NY, (-26.1833, -58.1833**)**; *Guaycolec* – [8,h], H. Maturo, D. Prado, SI 53, 20/09/2004, H. Maturo det., (-25.9845, -58.1614); *Patiño* – [8,h], P. Arenas, SI 1961,, 18/01/1982, P. Arenas det., (-25.3333, -59.6833); **Jujuy:** *Cachipunco* – [8,g*], Rotman A., SI 898, 10/12/1983, (-24.4766, -64.535); *Coiruru* – [1,g], Silva-Luz, CL; Luz, LF 290, SPF 212454, 27/03/2014, Silva-Luz, CL det., (-23.8765, -65.4589); *Rio San Francisco* – [3,g], Venturi 9735, CAS, 19/10/1929, (-23.3041, -64.0791); *Santa Barbara* – [3,g], Fries 293, US, 09/07/1901, (-24.3086, -64.6603); *Sierra de Zapla* – [8,n], A. L. Cabrera, S. Botta, A. M. Cialdella, A. Rotman, SI 32003, 14/11/1980, Cabrera A. L. det., (-24.1833, -65.0666); *Tilcara* – [1], Kiesling, R; et al. 1157, SP 250141, (-

23.5772, -65.3936); Tumbaya – [7,g], A. Burkart et al. 30524, SI, 7/11/1974, (-23.8527, -65.4661); *Valle Grande* – [2], Cabrera 27874, SI, 14/09/1976, (-23.4755, -64.9469); **La Pampa:** *General Pico* – [8,f], A. Buckart, SI 9847, 10/11/1939, (-35.6666, -63.7333); *Santa Rosa* – [7,h], H. Schwabe, H. Fabris, LP 2123, 28/11/1959, (-36.6205, -64.3075); *Telén* – [3,f], Cabrera 4367, GH, 16/03/1938, (-36.2667, -65.5); *Victorica* – [9], Pérez Moreau – Perrone, BA 30107, 13/01/1951,(-36.2167, -65.434); **La Rioja:** *General Belgrano* – [7,h], G. Covas 1208, LP 45082, 25/02/1941, F. A. Barkley det., (-30.6316, -66.2663); *Miranda* – [8,n*], A. Buckart, SI 12450, 26/02/1941, F. A. Barkley det., (-29.3333, -67.6833); **Mendoza:** *Cacheuta* – [4*], GSB, 19/07/2014, (-33.0858, -69.0786); *Cuesta Cerrillos* – [4*], GSB, 30/10/2012 (-33.1224, -68.9171)*; La Paz* – [7], E. L. Gautier, A. L. C. det., (-33.4666, -67.55)*; Las Heras* – [4*], GSB, 26/11/2011 (-32.8507, -68.8404); *Lujan del Cuyo* – [4*], GSB, 13/02/2012 (-33.0339, -68.8822); *Maipu* – [7,d], Carette, Ruiz, LP 3749, 25/01/1936, F. A. Barkley det., (-32.9666, -68.75); *Malargüe* – [8,d], A. Prina, G. Alfonso, E. Morici, W. Muiño, SI 2349, 12/12/2003, (-35.4744, -69.5852); *Manzano Historico* – [4*], GSB, 30/10/2012 (-33.5979, -69.3823); *San Carlos* – [1,d], Silva-Luz, CL; Luz, LF 266, SPF 212449, 17/03/2014, Silva-Luz, CL det., (-33.8156, -69.1815); *San Rafael* – [2*], Lourteig 763, LIL, (-34.6, -68.3333); *Tunuyan* – [7,h], LP 2111, 29/01/1934, F. A. Barkley det., (-33.5666, -69.0166); **Missiones:** *Candelaria* – [6,a], Rodríguez, F. M. 51, SI045504, 02/02/1920, (-27.45, -55.7333); *General Manuel Belgrano* – [1,l], Keller, H.A. 3640, HUEFS 133311, 27/08/2006, (-26.25, -53.65); *Loreto* – [2], Rodriguez 244, SI, 27/03/1910, (-27.3319, -55.5231); *San Ignacio* – [2], Schwarz 2019, LIL, 04/02/1946, (-27.2583, -55.5392); *Santa Ana* – [3,a], Rodriguez 51, F, 03/02/1930, (-27.3694, -55.5822); **Neuquén:** *Senillosa* – [8,d], Sede, S. M., SI 288, 04/01/2012, (-39.01, -68.43)**;** **Rio Negro:** *General Roca* – [3,d], Fischer 11, F, 09/1914, (-39.0333, -67.5833); *San Antonio Oeste* – [8,d], Eskuche, Klein, SI 166, 17/01/1968, (-40.73, -64.9388); *San Carlos de Bariloche* – [7], Stuessy, Crawford, Crisci, Cigliano, Gentilli, LP 6746, 02/12/1984, (-41.1436, -71.2908); **Salta:** *Alemanía* – [3,g], 9830, GH, 27/11/1929, (-25.6238, -65.6133); *Anta* – [1,n], Ragonese 362, SPF 214447, 06/1934, F. A. Barkley, det., (-24.1136, -64.1247); *Cafayate* – [2], R. Winkler, LIL 277100, (-26.0833, -65.9666); *Campo Duran* – [3,g], Parodi 9171, GH, 23/01/1930, (-22.2333, -63.7); *Chicoana* – [1,g], Paula-Souza, J.; Ferrucci, M.S.; Rando, J.G.; Meza Torres, E.I. 7780, ESA056590, 25/01/2007, (-25.1, -65.55); *Cuesta del Ovispo* – [4*], GSB, F. Navarro, H. Beccacece, 19/04/2010 (-25.1663, -65.7338); *La Candelaria* – [6,n], Venturi, S. 5494, SI033347, 24 /07/1927, Múlgura de Romero, M. E, det., (-26.1, -65.1); *Las Juntas* – [3,i], Bruch 2874, NY, 15/12/1926, (-28.1333, -65.9); *Lumbreras* – [1,n], Ragonese 357, SPF 214448, 08/1934, F. A. Barkley, det., (-25.2, -64.9166); *Rosario de Lerma* – [7,f], M. M. Job, LP 1559, 01/1937, Barkley det., (-24.9833, -65.5833); *Rosario de la Frontera* – [2], Villa Carenzo 2696, LIL, (-25.7983, -64.9741); *San Ramón de la Nueva Orán* – [1,g], Mello-Silva, R; Forzza, RC; Marcato, AC 1908, SPF 159369, 13/12/2001, Novara, LJ det., (-23.9, -64.3383); *Tanti* – [3,g], Venturi 9573, GH, 20/09/1929, (-31.3333, -64.6); **San Juan:** *Calingasta* – [2], Castellanos, LIL 61827, 27/01/1950, (-31.336, -69.42); Jáchal – [8*], Tombesi, T. S. et al., SI 72, 16/02/2000, (-30.2333, -68.75); *Valle Fertil* – [2*], Cuezzo 2941, LIL, (-30.9080, -67.285); **San Luis:** *Alto Pencoso* – [3,h], Bruch-Carette, NY?, 02/1914, (-33.43, -66.93); *Lujan* – [1,n], Silva-Luz, CL; Luz, LF 277, SPF 212460, 22/03/2014, Silva-Luz, CL det., (-32.3708, -65.9269); *Nueva Galia* – [3,h], Rodriguez 1468, F, 24/11/1915, (-35.1166, -65.25); *Pedernal* – [7,f], A. P. Rodrigo. LP 2006, 11/1941, (-31.9833, -68.7333); *San Luis* – [1,h], Silva-Luz, CL; Luz, LF 276, SPF 212459, 22/03/2014, Silva-Luz, CL det., (-33.0388, -66.3233); limite *San Luis* – *La Pampa* – [8,d], Cordo, Ferrer, SI 77-d-48, 24/11/1977, (-35.0947, -65.1066); **Santa Cruz:** *Cerro Alto* – [2], José Steinbach, SI, 21/12/1921, (-48.65, -69.6166); *Lago Ghio* – [8,d], Zuloaga, F. O., SI 14771, 21/11/2013, C. Zanotti det., (-47.2836, -71.525); *Puerto Deseado* – [2], Odonell 3579, LIL, (-47.75, -65.9166); **Santa Fe:** entre *Vera* y *Margarita* – [7,f], Ragonese, LP 2913, 19/11/1938, F. A. Barkley, det., (-29.5847, -60.2308); *Lago Argentino* – [6,a], Molina Massey, E. 48, SI033226, 11/1909, Múlgura de Romero, M. E. det., (-50.0702, -72.1794); *Lago Cardiel* – [6,a], Scott de Birabén, María Isabel, Birabén, Max 150, LP005406, 27/02/1936, Angel Lulio Cabrera det., (-48.95, -71.2166); *Lanteri* – [3,f], Job 1256, NY, 01/01/1936, (-28.8333, -59.65); *Reconquista* – [6,j], Kermes LP011017, 1900, F. A. Barkley det., (-29.2333, -59.9333); *Villa Guillermina* – [7,f],

Ragonese, LP 2920, 14/11/1938, F. A. Barkley, det., (-28.2403, -59.4667); *Tostado* – [1], Krapovickas, A; Vanni, R 43685, UB, 30/03/1990, (-29.2263, -61.7719); *Villa Ocampo* – [3,f], Venturi 153, FAB/NY, (-28.4833, -59.35); **Santiago del Estero:** Choya – [7], R. Maldonado Bruzzone, LP 1525, 27/09/1944, (-28.495, -64.8569); *Jiménez* – [3,g], 10113, GH, 16/02/1930, (-27.1633, -64.4908); *Moreno* – [2], Castellanos, LIL 268977, (-27.3702, -62.2655); *Ojo de Agua* – [7], Maldonado Bruzzone, LP 1504, 27/07/1944, (-29.5019, -63.6925); *Parque Nacional Copo* – [8,h], J. P. Pelotto, SI 3, 20/03/1992, (-25.7833, -62.0666); *Pellegrini* – [3,g], Venturi 5775, CAS, 22/12/1927, (-26.2, -64.2411); *Santiago del Estero* – [2], Juan Medina, LIL 514396, 28/09/1958, (-27.7844, -64.2669); **Tucumán:** *Burruyacu* – [2], Villa Carenzo, LIL,16/09/1961, (-26.4980, -64.7411); *El Mollar* – [2], Descole 1405, LIL, (-26.95, -65.7166); *El Siambón* – [4*], GSB, 17/07/2014, 27/08/2014, 16/03/2015, 23/11/2015 (-26.6866, -65.4447); *Leales* – [2], Krapovickas 1728, LIL, 12/02/1945, (-27.0327, -65.3072); *Los* Puestos – [7], A. Krapovickas, C. L. Cristóbal, LP 17392, 01/01/1971, (-27.2811, -65.0189); *Lules* – [3,g], 2291, GH, 14/10/1923, (-27.0220, -65.2640); *San Miguel de Tucuman* – [2], Venturi 3800b, LIL, (-26.8166, -65.2166); *Tafi Viejo* – [3], Lorentz, GH, 11/03/1879, (-26.7325, -65.2670); *Tapia* – [2], Rodrigues 10, SI, 18/09/1929, (-26.5880, -65.27972); *Trancas* – [2], Barkley 41505, LIL, (-26.2308, -65.2780). BOLIVIA: **Cochabamba:** *Misque* – [3], Cardenas 2138, GH, 06/1940, (-17,9427, -65.3416); *Quillacollo* – [3,l], Kuntze, NY, 4/05/1892, (-17.2855, -66.3916); **Hernando Siles:** *Monteagudo* – [1,g], Beck, SG 33614, SPF 215067, 25/12/2011, Silva-Luz, CL det., (-19.7883, -63.9436); **La Paz:** *Larecaja* – [6,c], Mandon, G. 768**,** K000537442, 1898, Muñoz, J.D. det., (-15.8333, -68.1666); **Santa Cruz:** *Camiri* – [1,g], Silva-Luz, CL 239, SPF 215058, 08/01/2014, Silva-Luz, CL det., (-19.9747, -63.5445); *Samaipata* – [1,h], Silva-Luz, CL 230, SPF 215069, 06/01/2014, Silva-Luz, CL, det., (-18.1738, -63.8782); Trigal – [1,h], Silva-Luz, CL 232, SPF 215049, 06/01/2014, Silva-Luz, CL det., (-18.3675, -64.1570); **Tarija:** *Tarija* – [2], T. Mayer, Legname, Cuezzo 22999, LIL 62642, 09/02/1970, (-21.5316, -64.7311). BRAZIL: **Distrito Federal:** *Brasília* – [6,c], Sellow, Friedrich, S10-18344, Muñoz, J.D. det., (-15.7938, -47.8827); **Espírito Santo:** *Venda Nova do Imigrante* – [1], Liebsch, JOI 8186, 07/08/2006, (-20.3397, -41.1347); **Mato Grosso do Sul:** *Antônio João* – [1,a], C. L. Silva-Luz 312, NY 02423642, 11/12/2014, C. L. Silva-Luz det., (-22.3134, -55.7490); *Bela Vista* – [1], Hatschbach, G. 74563, HUEFS 133171, 11/03/2003, S.F.Smith det., (-22.1089, -56.5211); *Bonito* – [1], Barbosa, E; Silva, JM 1965, MBM 323112, 18/06/2006, SF, Smith det., (-21.1236, -56.4922); *Dourados* – [1], Fujii, G.; Magenta, M.A.G.; Martins, S.E.; Abolis, D. 1, HUSC 8288, 07/05/2008, (-22.2230, -54.8302); *Porto Murtinho* – [1,a], Ratter, J. A.; Pott, A.; Bridgewater, S.; Fonseca Filho, J. 7534, CPAP 19074, 26/09/1996, (-21.5, -56.5); *Rio Brilhante* – [1,a], G. G. Hatschbach 26178, NY 1030776, 27/01/1971, J. de D. Muñoz det., (-21.8019, -54.5464); **Minas Gerais:** *Alagoa* – [1], Giacomin, LL; Viana, PL; Oliveira, CT; Perillo, LN 1077, BHCB 132862, 20/08/2009, (-22.1706, -44.6419); *Camanducaia* – [1*], Kinoshita, L.S. s.n., UEC 125737, 17/05/2002, Meireles, L.D det., (-22.7553, -46.1447); *Delfim Moreira* – [1], L.N.Gonçalves, N.M.Ivanauskas & R.T.Polisel, M.G.K.Cornia 314, SPSF 48259, 17/02/2014, R.T.Polisel det., (-22.5294, -45.2797); *Gonçalves* – [1], Kamino, LHY; França, GS; Stehmann, JR 94, BHCB 53524, 24/08/2000, (-22.6589, -45.8558); *Maria da Fé* – [1,k], A. P. Duarte 279, NY 1104795, 30/08/1946, (-22.3083, -45.3753); **Paraná:** *Almirante Tamandaré* – [11], Moreira, G.R.P., Carneiro, E., Zacca, T. & Basilio, D. legs., 21/03/2013, LMCI 213, (-25.3193, -49.3041); *Boa Ventura de São Roque* – [1], Lozano, ED; Canestraro, BK 1033, MBM 382811, 17/09/2012, Lozano, ED det., (-24.9336, -51.6422); *Bituruna* – [1,b], Hatschbach, G 14971, SPF 32711, 18/05/1984, Pirani, JR det., (-26.1614, -51.5525); *Campo Largo* – [11], Moreira, G.R.P., Carneiro, E., Zacca, T. & Basilio, D. legs., 21/03/2013, LMCI 214 (-25.4595, -49.5272); *Campo Mourão* – [1,e], A.E. Bianek 16, HCF 165, 09/05/2003, G. Hatschbach det., (-24.0456, -52.3831); *Carambei* – [1], Engels, ME; Imig, DC 1452, MBM, 10/08/2013, Barboza, E det., (-26.3040, -52.0109); *Castro* – [1], Hatschbach, G 32313, MBM 28031, 14/08/1973, Hatschbach, G det., (-24.7908, -50.0138); *Colombo* – [1,j], Pegoraro, A 123, MBM 144309, 02/09/1987, Hatschbach, G det., (-25.2919, -49.2238); *Contenda* – [1,j], Hatschbach, G., UPCB 3737, 09/1962, Hatschbach, G., (-25.6756, -49.5347); *Coronel Domingos Soares* – [1], Motta, JT; et al. 4101, ICN 179488, 2/09/2013, Cordeiro, J det., (-26.3041,

-52.0108); *Curitiba* – [11], 22/02/2008, Moreira, G.R.P.. Ribas, O.S., Carneiro, E., Beltrami, L. legs., LMCI 14, (-25.4288, -49.2671); *Fazenda Rio Grande* – [1], Dunaiski Jr, A 1294, MBM 390933, 28/08/1999, Cordeiro, J; Barboza, E det., (-25.6611, -49.3088); *General Carneiro* – [1], Hatschbach, G 14990, MBM 1831, 18/10/1966, Pirani, JR det., (-26.4275, -51.3156); *Guarapuava* – [1], Hatschbach, G; Guimarães, O 19877, MBM 15684, 27/09/1968, Hatschbach, G det., (-25.3953, -51.4581); *Inácio Martins* – [1], Hatschbach, G. 72441, HUEFS 64846, 20/09/2001, (-26.4166, -51.3); *Lapa* – [1], Oliveira, PI 619, MBM 84351, 13/08/1982, Hatschbach, G det., (-25.7675, -49.7172); *Mandirituba* – [1], Dunaiski Jr., A. 143, UPCB 24639, 15/09/1991, Guimarães, O.A. det., (-25.7789, -49.3261); *Mangueirinha* – [1,e], Silva, JM; Baigerl, IG 8921, ICN 193016, 29/07/2015, Barboza, E det., (-26.0472, -52.1783); *Palmas* – [1], Hatschbach, G. 72386, UPCB 45154, 19/09/2001. Hatschbach, G.det., (-26.4842, -51.9906); *Piraí do Sul* – [1], Souza, LRM; et al. s.n., FUEL 26196, 22/07/1999, (-24.5261, -49.9486); *Piraquara* – [1], Brotto, ML; Barboza, E; Cordeiro, J; Silva, JM 1393, MBM, 24/10/2013, Brotto, ML det., (-25.4498, -49.0144); *Pitanga* – [1], Bianek, A.E. 315, HUTO 4806, 05/10/2007, Caxambu, M.G.det., (-24.7572, -51.7614); *São Jose dos Pinhais* – [1], Miranda, A.C.L. 251, IRAI 1314, 26/09/2009, Cordeiro, J. det., (-25.5329, -49.1634); *Turvo* – [1], Caxambu, M.G.; et al. 2662, DVPR 3284, 06/08/2009, Caxambu, M.G.det., (-25.02, -51.33); **Rio de Janeiro:** *Rio de Janeiro* – [1], Glaziou, A. 19606, P06634234, 19/9/1890, (-22.9028, -43.2075); **Rio Grande do Sul:** *Alegrete* – [1], Marchiori, J. N. C. 765, HDCF 3137, 30/03/1987, Figueira, M. det., (-29.7831, -55.7919); *Arroio dos Ratos* – [2], M. Fleig 605, ICN, 23/10/1976, (-30.0769, -51.7288); *Bagé* – [11], 02/05/2014, Moreira, G.R.P. & Vaz, C.M.L. legs., LMCI 268, (-31.3283, -54.0336); *Bom Jesus* – [1], Wasum, R. 2309, HUCS 25884, 7/01/2005, Sobral, M. det., (-28.6678, -50.4167); Bossoroca – [1], Bamberg, R. s.n., HDCF 6621, 28/05/2013, Figueira, M. det., (-28.73, -54.9003); Caçapava do Sul – [1], Schlindwein, C., MPUC 12826, 21/01/1993, Schlindwein, C. det., (-30.5125, -53.4913); *Cachoeira do Sul* – [2], O. R. Camargo, HAS 109, 16/08/1975, (-30.0388, -52.8938); *Camaquã* – [2], M. Fleig 805, ICN, 10/10/1977, (-30.8508, -51.8119); *Cambará do Sul* – [1*], Irgang, BE; Baptista, LRM s.n., FLOR 26738, 26/07/1978, Irgang, BE; Baptista, LRM det., (-29.0478, -50.1447); Campestre da Serra – [11], Moreira, G.R.P. & Gonçalves, G.L. legs., 18/01/2013, LMCI 207, (-28.7930, -51.0946); *Canela* – [1*], Adamy, S. s.n., HDCF 3985, 02/1989, Sobral, M. det., (-29.3656, -50.8156); *Canguçu* – [11], 08/04/2012, Luz, F.A. leg., LMCI 175, (-31.3965, -52.6786); *Canoas* – [1], N. Rolim Bastos 83, PACA 70132, 07/05/1989, D. Tobolski det., (-29.9178, -51.1836); *Capão do Leão* – [1], Jarenkow, JA 2563, MBM 207128, 17/03/1995, Jarenkow, JA det., (-31.7852, -52.5025); *Caxias do Sul* – [1*], Beltrão, R. 33, HUCS 35594, 14/08/2009, Link, O. det., (-29.1681, -51.1794); *Condor* – [2], B. Irgang, ICN 4751, 25/03/1967, (-28.2077, -53.4869); *Coxilha* – [1], Marchett, F. 383, HUCS 26690, 29/09/2005, Smith, S..F. det., (-28.1272, -52.2961); *Dom Pedrito* – [1], M. Rossato 311, JOI 15101, 12/04/2005, F. Gonzatti det., (-30.9847, -54.6366); *Dom Pedro de Alcântara* – [1], Mondin, C. et all., MPUC 11918, 22/03/2009, C, Mondin det., (-29.3694, -49.8497); *Encruzilhada do Sul* – [1*], Pastore, U 15, FLOR 8286, 25/10/1980, Klein, RM det., (-30.5439, -52.5219); *Esmeralda* – [1*], Mauhs, J., PACA 94196, 07/08/2002, J. Mauhs det., (-28.0536, -51.1903); *Estação* – [1], Butzke, A.; et al s.n., HUCS 11553a, 24/08/1995, Smith, S. F.det., (-27.9108, -52.26); *Esteio* – [1], Rambo, B. 40,61, E00089933, 23/03/1949, (-29.8613, -51.1791); *Farroupilha* – [1], Aguiar, L. s.n., HAS 8457, 08/1978, Bueno, O. det., (-29.225, -51.3478); *Fortaleza* – [1,e], Ronaldo Wasum|et al. Wasum 12157, MO 2869600, 1997, Stephen F. Smith det., (-28.7969, -53.2227); *Giruá* – [1], Hagelund, K 5380, ICN 128138, 11/08/1967, Fleig, M det., (-28.0283, -54.3497); *Gravataí* – [1], Martau, L; et al. s.n., SP 249684, 06/06/1978, Bueno, O det., (-29.9444, -50.9919); *Guaíba* – [1], Torgan, L. s.n., HAS 4102, 04/1976, Bueno, O. det., (-30.1139, -51.325); *Itaquí* – [2], M. Fleig 552, ICN, 05/04/1977, (-29.125, -56.5527); *Jaquirana* – [1], Marchett, F. 647, HUCS 32039, 28/10/2007, Sobral, M., det., (-28.8847, -50.3578); *Júlio de Castilhos* – [1], Marchiori, J. N. C. s.n., HDCF 891, 29/10/1981, Longhi, S. J.; Marchiori, J. N. det., (-29.2269, -53.6817); *Lagoa Vermelha* – [2], M. Fleig 929, ICN, 06/01/1978, (-28.2088, -51.5258); *Lavras do Sul* – [2], M. Fleig 162, ICN, 12/12/1976, (-30.8127, -53.895); *Machadinho* – [1], Witt, A. s.n., HUCS 17886, 05/08/2000, (-27.5669, -51.6678); *Maximiliano de Almeida* – [1], Lutkemeier, C s.n., ICN 119811,

19/08/2000, (-27.6322, -51.8033); *Montenegro* – [1,b], E. Friderichs, PACA 34303, 11/1946, Emrich-Rambo det., (-29.6886, -51.4611); *Nonoai* – [1,b], Rambo, B., PACA 28540, 03/05/1954, Emrich-Rambo det., (-27.3617, -52.7714); *Nova Prata* – [1], Giacomolli, L. Z. s.n., HDCF 5647, 07/09/2005, Longhi, S. J. det., (-28.7839, -51.61); *Osório* – [1], Gonzatti, F. 1889, HUCS 42677, 21/04/2015, Gonzatti, F. det., (-29.9577, -50.9581); *Pantano Grande* – [1], Scipioni, M. C.; Longhi, S. J. s.n., HDCF 6124, 29/04/2008, Longhi, S. J.; Scipioni, M. C. det., (-30.1128, -52.3805); *Pareci Novo* – [1,c], Henz, E., PACA 27578, 1944, Emrich-Rambo det., (-29.6377, -51.3977); *Passo Fundo* – [1], Longhi, S. J. 315, HDCF 1079, 22/03/1982, Longhi, S. J. det., (-28.3166, -52.1844); *Pelotas* – [1], T.S. Venske 80, ECT 51, 23/05/2008, T.S. Venske det., (-31.7138, -52.1791); *Pinheiro Machado* – [1], Rossato, M. 309, HUCS 26714, 12/04/2005, Smith, S.F. det., (-31.5783, -53.3811); *Piratini* – [2], M. Fleig 794, ICN, 09/10/1977, (-31.4477, -53.1038); *Porto Alegre* – [1], Rambo, B. s.n., SMDB 54, 17/03/1933, Záchia, R.A det., (-30.0331, -51.23); *Quaraí* – [1], Rossato, M. 318, HUCS 31136, 14/04/2005, Gonzatti, F. det., (-30.4016, -56.1850); *Restinga Seca* – [2], M. Fleig 989, ICN, 19/03/1978, (-29.8127, -53.375); *Rio Grande* – [1], Coura Neto, AB; Moreno, JA 56, FLOR 8341, 31/10/1980, Falkenberg, DB det., (-32.035, -52.0986); *Rio Pardo* – [2], M. Fleig 256, ICN, 01/01/1976, (-29.99, -52.3777); *Rosário do Sul* – [2], J. Waechter & M. L. Porto 263, ICN, 29/05/1976, (-30.2577, -54.9138); *Santa Maria* – [1], Beltrão, R. s.n., SMDB 687, 05/01/1952, Beltrão, B det., (-29.6842, -53.8069); *Santa Rosa* – [1], Hagelund, K 5378, ICN 128134, 13/08/1967, (-27.8708, -54.4814); *Santana do Livramento* – [1], Pirani, JR; Yano, O 513, SP 183790, 22/01/1983, Pirani, JR det., (-30.8908, -55.5328); *Santiago* – [2], M. Fleig 113, ICN, 10/12/1976, (-29.1919, -54.8669); *Santo Ângelo* – [2], M. Fleig 83, ICN, 09/12/1976, (-28.2988, -54.2627); *Santo Antônio da Patrulha* – [1], Sangoi Rodrigues, T. E. s.n., HDCF 5957, 16/03/2006, Sangoi Rodrigues, T. E. det., (-29.8175, -50.5197); *São Borja* – [1], Záchia, R 826, MBM 181289, 17/01/1992, Mondin, C det., (-28.6829, -55.9775); *São Francisco de Assis* – [2], M. Fleig 133, ICN, 10/12/1976, (-29.55, -55.1308); *São Francisco de Paula* – [11], 29/11/2011, G.R.P. Moreira leg., LMCI 165, (-29.4460, -50.5806); *São Gabriel* – [1,a], Marchiori, J. N. C. 278, HDCF 2230, 15/01/1986, Marchiori, J. N. C. det., (-30.3364, -54.32); *São Joao do Polêsine* – [1,c], Sehnem, A. 1334, PACA 104395, 09/04/1956, A. sehnem, det., (-29.6138, -53.4458); *São Jorge* – [1,l], Pasini, E. 264, HUCS 35483, 21/07/2009, Link, O det., (-28.5006, -51.70); *São José dos Ausentes* – [4*], Lúcia Sevegnani s.n., MBM 318722, 4/5/1997, (-28.7477, -50.0658); *São Leopoldo* – [1,c], Henz, E., PACA 33528, 16/04/1946, Emrich-Rambo det., (-29.7603, -51.1472); *São Luiz Gonzaga* – [1,c], Rambo, B., PACA 53312, 24/11/1952, Emrich-Rambo det., (-28.4077, -54.9608); *São Marcos* – [1], Rossi, T 28, FUEL 49893, 31/08/2011, Grizzon, M, det., (-28.9711, -51.0681); *São Sepé* – [1], Longhi, S. J.; Marchiori, J. N. C. s.n., HDCF 603, 01/07/1982, Longhi, S. J. det., (-30.1606, -53.5653); *Silveira Martins* – [1,c], Pivetta, J. 1334, HRCB 44116, 09/04/1956, Rambo det., (-29.6425, -53.5856); *Tainhas* – [4*], A. R. Cordeiro, ICN 2813, 14/11/1961, M. Fleig det., (-29.2741, -50.3130); *Tapes* – [2*], M. Fleig 813, ICN, 10/10/1977, (-30.6727, -51.3958); *Torres* – [1], Hagelund, K 5119, ICN 153353, 15/01/1967, Fleig, M det., (-29.3353, -49.7269); *Triunfo* – [1], Ungaretti, I. 175, HAS 4415, 04/1977, Bueno, O. det., (-29.9433, -51.7181); *Tupanciretã* – [1], Wasum, R.; Bastos, N. s.n., HUCS 12563, 11/11/1991, Smith, S..F. det., (-29.0806, -53.8358); *Uruguaiana* – [1], Longhi, S. J. 817, HDCF 2645, 31/08/1987, Longhi, S. J., (-29.7547, -57.0883); *Vacaria* – [11], 18/01/2013, Moreira, G.R.P. & Gonçalves, G.L. legs., LMCI 206, (-28.5016, -50.9340); *Venâncio Aires* – [2], M. Fleig 971, ICN, 18/03/1978, (-29.6058, -52.1919); *Viamão* – [1], Wiesbauer, MB 60, ICN 132355, 03/04/2004, Grings, M det., (-30.0811, -51.0233); **Santa Catarina:** *Abelardo Luz* – [2], R.M. Klein 5530, HBR, 26/08/1964, (-26.565, -52.3277); *Agua Doce* – [4*], Smith, LB; Klein, RM 13568, FLOR 2822, 04/12/1964, Falkenberg, DB det., (-26.6833, -51.6166); *Anita Garibaldi* – [1*], Stival-Santos, A. 262, FURB 465, 12/12/2008, Sobral, M det., (-27.7175, -51.0286); *Araranguá* – [1], Citadini-Zanette 2051, CRI 109, 30/06/1996, Daniel B. Falkenberg det., (-28.9352, -49.4861); *Bom Jardim da Serra* – [1], Cervi, A.C. 10193, HUEFS 210772, 07/11/2013, (-28.3355, -49.68); *Bom Retiro* – [1,b], Rambo, B., PACA 60129, 22/01/1957, Emrich-Rambo det., (-27.7972, -49.4892); *Campo Alegre* – [2], R. Reitz & R.M. Klein 4051, HBR, (-26.1927, -49.2658); *Campo Belo do Sul* – [1], Buzatto, CR 350, ICN 189629, 19/10/2007,

Jarenkow, JÁ det., (-27.8992, -50.7608); *Campos Novos* – [2], R. Reitz & R.M. Klein 15108, HBR, 09/07/1963, (-26.1927, -49.2658); *Canoinhas* – [2], R.M. Klein 3021, HBR, 15/09/1962, (-26.1769, -50.39); *Celso Ramos* – [1], A. Korte 7076, JOI 11986, 18/07/2011, A. Korte det., (-27.7155, -51.2988); *Curitibanos* – [11], 17/01/2013, Moreira, G.R.P. & Gonçalves, G.L. legs., LMCI 205, (-27.2828, -50.5808); *Itaiópolis* – [1,b], Silva-Luz, CL 333, SPF 216471, 23/12/2014, Silva-Luz, CL det., (-26.3364, -49.9064); *Lages* – [1], Manfredi, S., LUSC 7829, 14/02/2011, (-27.8161, -50.3261); *Mafra* – [1], M. Verdi 5458, CRI 108, 20/08/2010, (-26.1119, -49.8055); *Painel* – [1], Ferreira, T.S. 1535, LUSC 2554, 1/09/2008, Higuchi, P. & Silva, A.C det., (-27.9289, -50.105); *Ponte Alta* – [2], R.M. Klein 2947, HBR, 13/09/1962, (-27.4838, -50.38); *Rancho Queimado* – [1], Reitz, R; Klein, RM 9704, FLOR 4, 11/08/1960, (27.6725, -49.0217); *São Bento do Sul* – [1], Schwirkowski, P., FPS 1410, 13/10/2013, Schwirkowski, P. det., (-26.2841, -49.4607); *São Cristóvão do Sul* – [11], 15/01/2013, Moreira, G.R.P. & Gonçalves, G.L. legs., LMCI 201, (-27.2671, -50.4392); *São Joaquim* – [11], 16/01/2013, Moreira, G.R.P. & Gonçalves, G.L. legs., LMCI 204, (-28.2926, -49.9378); *São José* – [1,j], R. Reitz 9704, NY 437976, 11/08/1960, L. B. Smith det., (-27.6156, -48.6272); *Sombrio* – [2], R. Reitz C532, HBR, 12/04/1944, (-29.1038, -49.6288); *Urubici* – [1], Negrini, M., LUSC 5717, 17/12/2011, Higuchi, P. det., (-28.015, -49.5917); *Urupema* – [1], Rodrigues, M., LUSC 3906, 02/11/2009, Rodrigues, C det., (-27.9528, -49.8731); *Vargem Bonita* – [1], Liebsch, D 1116, MBM 334483, 07/08/2006, (-26.9455, -51.7477); **São Paulo:** *Campos do Jordão* – [10], 30/05/2014, Moreira, G.R.P. & Nihei, S. legs., LMCI 270, (-22.7428, -45.5964); *Imbituva* – [1,j], José R. Pirani 935, MO 2868768, 1984, J.R. Piranidet., (-25.23, -50.6044); *São Bento do Sapucaí* – [11], 30/05/2014, Moreira, G.R.P. & Nihei, S. legs., LMCI 271, (-22.4114, -45.4355); *São Paulo* – [1,j], Queiroz, LP de; Martins, E; Carvalho-Okano, RM; Guimarães, PF; et al. 2683, ALCB 029408, 06/10/1990, (-22.7166, -45.5666); CHILE: **Araucania:** *Temuco* – [3], Claude-Joseph 4831, AHH, 10/1925, (-38.75, -72.6666); **Atacama:** *La Cebolla* – [3], Johnston 4852, GH, 02/11/1925, (-27.3666, -70.3333); **BioBío:** *Antuco* – [6], Gardner, Martin F. & Page, Christopher N. 5033, E00089924, 16/02/1992, (-37.3333, -71.6833); *Concepción* – [6,c], Bridges 1337, K000537428, Muñoz, J.D. det., (-36.8333, -73.05); **Coquimbo:** *Combarbala* – [6], Gardner, Martin F. & Knees, Sabina G.5628, E00089923, 26/02/1993, (-31.1680, -71.0086); *Ovalle* – [3], REW, GH, (-30.5833, -71.2); *Vicuña* – [3], Werderman 85, CAS, 11/1923, (-30.0333, -70.7); **Elqui:** *Coquimbo* – [1], Moreira, A s.n., SPF 212384, 20/09/2013, (-30.6408, -71.6502); **Los Rios:** *Mariquina* – [3], Hallermeyer (Werderman, 1191), AHH, 03/1926, (-39.5166, -72.9666); *Panguipulli* – [3], Claude-Joseph 2587, US, 07/1924, (-39.6444, -72.3305); **Maule:** *Cauquenes* – [3], Sargent, AHH, 02/1906, (-35.9669, -72.3258); *Romeral* – [3], Mexia 7870, F, 13/02/1936, (-34.9666, -71.1333); *San Clemente* – [1], Silva-Luz, CL; Luz, LF 198, SPF 212429, 22/09/2013, (-35.8255, -70.7547); **O'Higgins:** *Colchagua* – [3], Eduardo Moore, SGO, 1924, (-34.6833, -71.15); *Rio Claro* – [2], Ricardi, LIL 353754, 01/01/1951, (-34.8119, -70.7419); *San Gabriel* – [3], West 6010, UC, 07/02/1936, (-33.7833, -70.25); **Metropolitana de Santiago:** *Lo Barnechea* – [3], Werderman 486, CAS, 12/1924, (-33.35, -70.5166); *Renca* – [3], Montero 57, GH, 10/1922, (-33.4033, -70.7166); *Santiago* – [1], Silva-Luz, CL; Luz, LF 192, SPF 212400, 18/09/2013, (-33.4155, -70.6219); **Valdivia:** *S. José de la Mariquina* – [7], P. Ath. Hollermayer, LP 1191, 03/1926, (-39.5166, -72.9666); **Valparaiso:** *Alicahue* – [6], Gardner, Martin Fraser; Eski, Gülnur; Hepp, Josefina; Pestell, Jacqui 91, E00399956, 2010/11/02, (-32.3787, -70.8407); *Los Andes* – [3], Johnston 6275, GH, (-32.8166, -70.6166); *Quilpué* – [3],West 3972, UC, 28/11/1935, (-33.05, -71.45); *San Felipe* – [3], Claude-Joseph 3817, US, 12/1929, (-32.75, -70.7333); Valparaiso – [3], Cuming, NY, 1832, (-33.0406, -71.628); PARAGUAY: **Amambay:** *Pedro Juan Caballero* – [6,l], Hassler, E. 11261, 1912-1913, (-22.53, -55.75); **Presidente Hayes:** *Estancia Vanguardia* – [8,h], J. De Egea Juvinel, Ss. Centrón, SI 383, 30/03/2004, J. De Egea det., (-23.7691, -57.6872); *Isla Poi* – [7], T. Rojas, LP 7082, 08/1934, (-22.5297, -59.7277); URUGUAY: **Artigas:** *Arroyo Itacumbú* – [10,a], Del Puerto, MVFA 11384, 11/10/1972, (-30.3655, -57.6582); *Artigas* – [1], Longhi, S. J. s.n., HDCF 4550, 30/10/1991, (-30.4666, -56.4666); *Tres Cerros de Catalán* – [1], Longhi, S. J. s.n., HDCF 4588, 30/10/1991, (-30.6669, -56.5000); **Canelones**: *Los Cerrillos* – [10,j], E. H.Marchesi, MVFA 219, 21/09/1962, (-34.6072, -56.3597); *Santa Lucia* –

[3,a], Safford, US,01/11/1886, (-34.4525, -56.3963); *Toledo* – [1,a], BCTw 10393, (-34.7433, -56.1041); **Cerro Largo:** *Melo a Rio Branco* – [10,a*], Alano, Ziliani, Colero, Amás, MVFA 8742, 20/10/1969, (-32.5969, -53.385)*; Sierra de Ríos* – [6,a], Herter, W.G.F. 1871, SI033223, 02/1937, Múlgura de Romero, M. E. det., (-32.1163, -53.9991); **Colonia:** *Juan Jackson* – [6,j], Gallinal; Aragone; Bergalli; Campal; Rosengurtt, Bernardo PE - 4321 1/2, LP011003, 09/1940, F. A. Barkley det., (-33.9165, -57.2319); *Nueva Palmira* – [9,j*], M. Doello Jurado, BA 1780, 09/04/1927, F. A. Barkley det., (-33.8833, -58.2166); *Punta Gorda* – [10,a], Marchesi, Del Puerto, MVFA 8913, 30/10/1969, (-32.9161, -58.4144); **Florida:** *Casupá* – [10], Del Puerto, Borsani, MVFA 5566, 02/12/1965, (-34.1005, -55.6480); *Florida* – [2], Rosengurt, PACA 32951, (-34.1, -56.2166); *San Pedro del Timote* – [1,c], B. Rosengurtt, PACA 32951, 02/10/1945, B. Rosengurtt det., (-33.7880, -55.6713); **Lavalleja:** *Minas* – [1,a], Silva-Luz, CL; Luz, LF 219, SPF 212407, 27/11/2013, Silva-Luz, CL, (-34.1439, -54.9011); *Puente sobre el Cebollatí* – [10], Olano, Zilliani, Calero, Duran, MVFA 8680, 19/10/1969, (-33.8294, -54.7688); **Maldonado:** *Piriápolis* – [10,j], Del Puerto, Marchesi, MVFA 7689, 1/11/1968, (-34.8661, -55.2747); *Sierra de las Animas* – [10,j], Costa, MVFA 6712, 19/11/1967, (-34.7477, -55.3211); **Montevideo:** *Montevideo* – [10,h*], Arrillaga, MVFA 2576, 11/1968, (-34.8372, -55.2197)**; Paysandu:** *Chapicuy* – [10], Rosengurtt, MVFA 9281, 06/12/1962, (-31.6666, -57.8833); *Paysandu* – [1,a], Silva-Luz, CL; Luz, LF 215, SPF 212405, 24/11/2013, Silva-Luz, CL det., (-32.4649, -58.0166); *Saladero Guaviyú* – [10,a], Rosengurtt, Del Puerto, Marchesi, MVFA 10610, 17/01/1967, (-31.7638, -58.0391); **Rio Negro:** *Tres Bocas* – [10,o*], Marchesi, Bonifacino, MVFA 24481, 18/04/1995, E. H. Marchesi det., (-32.7669, -57.9205); **Rivera**: *Cuñapiru* – [1,c], Rambo, B., PACA 4019, 04/05/1954, Emrich-Rambo det., (-31.5730, -55.4791); *Massoler* – [10*], Del Puerto, Marchesi, MVFA 8612, 1/09/1968, (-31.0847, -56.0083); *Rivera* – [2], B. Rambo, PACA 4019, (-30.8986, -55.5388); **Rocha:** *Castillos* – [6,j], Kermes 13509, LP011002, 1929, F. A. Barkley det., (-34.1988, -53.8575); **San Jose:** *Balneario Arazatí* – [10,a], Zilliani-Ren, MVFA 16038, 31/03/1979,(-34.56, -56.9972); *Rincón del Pino* – [10,j*], J.Reu, MVFA 10881, 3/11/1971, (-34.5039, -56.8344)*; Sierra Mahoma* – [10,j], Del Puerto, MVFA 2171, 22/09/1962, (-34.15, -56.95); **Tacuarembó:** *Gruta de los Helechos* – [8,a], Dematteis, M.,Schinni, A., SI 1795, 22/02/2005, A. Schinini det., (-31.6827, -56.0202); *Valle Edén* – [10*], Del Puerto, MVFA 3923, 10/01/1965, (-31.8233, -56.1772); **Treinta y Tres:** *Río Tacuari* – [10], Del Puerto, Marchesi, MVFA 2305, 09/11/65, (-32.7703, -53.3097); *Santa Clara de Olimar* – [3,a], Herter f255, US, 11/1926, (-32.8333, -54.9080); *Treinta y Tres* – [1,c], Silva-Luz, CL; Luz, LF 221, SPF 212416, 27/11/2013, Silva-Luz, CL det., (-33.2308, -54.3822).
