# Supplementary material for: Phylogeography of the gall-inducing micromoth Eucecidoses minutanus Brèthes (Cecidosidae) reveals lineage diversification associated with the Neotropical Peripampasic Orogenic Arc
Source: PLoS One. 2018 Aug 8;13(8):e0201251. doi: 10.1371/journal.pone.0201251 (PMC6082564; doi:10.1371/journal.pone.0201251)
Supplement: S1 Table — (DOCX) [file pone.0201251.s004.docx]

**S1 Table.** Primers used in this study and PCR conditions.

| **Locus** | **Primer** | **Sequence (5´–3´)** | **Reference** | **Size (pb)** | **Annealing temp.** |
| --- | --- | --- | --- | --- | --- |
| *CO-I* | k698 | F: TACAATTTATCGCCTAAACTTCAGCC | Caterino & Sperling 1999 | 732 | 55 |
|  | Nancy | R: CCCGGTAAAATTAAAATATAAACT | Caterino & Sperling 1999 |  | 55 |
|  | Jerry | F: CAACATTTATTTTGATTTTTTGG | Caterino & Sperling 1999 | 831 | 52 |
|  | PatII | R: TCCATTACATATAATCTGCCATATTAG | Caterino & Sperling 1999 |  | 52 |
| *CO-II* | George | F: ATACCTCGACGTTATTCAGA | Caterino & Sperling 1999 | 750 | 47 |
|  | Eva | R: GAGACCATTACTTGCTTTCAGTCATCT | Caterino & Sperling 1999 |  | 47 |
| *16S* | 16Sar | F: CCCGCCTGTTTATCAAAAACAT | Palumbi (1996) | 990 | 55 |
|  | Ins16Sar | R: CCCTCCGGTTTGAACTCAGATC | Palumbi (1996) |  | 55 |

References:

Caterino MS, Sperling FAH. *Papilio* phylogeny based on Mitochondrial Cytochrome Oxidase I and II genes. Mol Phyl Evol 1999; 11: 122–137.

Palumbi, S.R. 1996. PCR and molecular systematics. In Molecular Systematics, 2nd edition,D. Hillis, C. Moritz, and B. Mable, Eds. Sinauer Press.
